# Natriuresis-guided diuretic therapy in acute heart failure: a pragmatic randomized trial

**DOI:** 10.1038/s41591-023-02532-z

**Published:** 2023-08-28

**Authors:** Jozine M. ter Maaten, Iris E. Beldhuis, Peter van der Meer, Jan A. Krikken, Douwe Postmus, Jenifer E. Coster, Wybe Nieuwland, Dirk J. van Veldhuisen, Adriaan A. Voors, Kevin Damman

**Affiliations:** 1 Department of Cardiology, University Medical Center Groningen, University of Gronigen, Groningen, the Netherlands; 2 Department of Epidemiology, University Medical Center Groningen, University of Groningen, Groningen, the Netherlands

**Keywords:** Heart failure, Outcomes research

## Abstract

Measurement of natriuresis has been suggested as a reliable, easily obtainable biomarker for assessment of the response to diuretic treatment in patients with acute heart failure (AHF). Here, to assess whether natriuresis-guided diuretic therapy in patients with AHF improves natriuresis and clinical outcomes, we conducted the pragmatic, open-label Pragmatic Urinary Sodium-based algoritHm in Acute Heart Failure trial, in which 310 patients (45% female) with AHF requiring treatment with intravenous loop diuretics were randomly assigned to natriuresis-guided therapy or standard of care (SOC). In the natriuresis-guided arm, natriuresis was determined at set timepoints, prompting treatment intensification if spot urinary sodium levels were <70 mmol l^−1^. The dual primary endpoints were 24 h urinary sodium excretion and a combined endpoint of time to all-cause mortality or adjudicated heart failure rehospitalization at 180 days. The first primary endpoint was met, as natriuresis in the natriuresis-guided and SOC arms was 409 ± 178 mmol arm versus 345 ± 202 mmol, respectively (*P* = 0.0061). However, there were no significant differences between the two arms for the combined endpoint of time to all-cause mortality or first heart failure rehospitalization, which occurred in 46 (31%) and 50 (31%) of patients in the natriuresis-guided and SOC arms, respectively (hazard ratio 0.92 [95% confidence interval 0.62–1.38], *P* = 0.6980). These findings suggest that natriuresis-guided therapy could be a first step towards personalized treatment of AHF. ClinicalTrials.gov registration: NCT04606927.

## Abstract (web summary)

In patients with acute heart failure, personalized dosing of a diuretic led to treatment intensification in the majority of patients and improved natriuresis, but had no effects on time to all-cause mortality or heart failure rehospitalization.

## Main

Acute heart failure (AHF) is one of the leading causes of hospitalization in the world and associated with high morbidity and mortality^1,2^. The main treatment goal in patients presenting with AHF is to reach euvolemia by the use of decongestive therapies, mainly loop diuretics^3^. There is, however, great variation in how monitoring of diuretic response is handled after admission. Often surrogate measures of diuretic response are used such as weight loss, which have been shown to be insensitive, often inaccurate and slowly affected^4–6^. Furthermore, a large number of AHF patients display an insufficient diuretic response even early after the start of loop diuretic therapy, which is associated with residual congestion and an increased risk of mortality and heart failure (HF) rehospitalization^5–7^. Given the mode of action of loop diuretics, assessment of natriuresis could not only be a sensitive marker to assess response, but also be a potential treatment target to guide decongestive therapy. Several observational studies have shown that insufficient natriuresis following loop diuretic administration is associated with poor diuretic response and an increased risk of all-cause mortality and HF rehospitalization^8–10^. Additionally, greater sodium excretion and a net negative sodium balance have been shown to be associated with better clinical outcomes, whereas net negative fluid balance was not^9^. So far, limited, nonrandomized data suggest using natriuresis as a marker to guide decongestive therapy in patients with AHF might be useful to improve diuretic response (Protocolized natriuresis-guided decongestion improves diuretic response: the multicenter ENACT-HF study. Dauw, J. et al., submitted)^11^. Despite this, current European Society of Cardiology (ESC) HF guidelines already suggest the early and repeated assessment of spot urinary sodium in patients admitted with AHF to guide diuretic treatment^3^. Therefore, there is need for trial data to support these recommendations, and to provide randomized evidence on the use of a reliable, easily obtainable, inexpensive, readily available and implementable marker to assess response and guide treatment in patients with AHF. In this article, the Pragmatic Urinary Sodium-based algoritHm in Acute Heart Failure (PUSH-AHF) randomized clinical trial investigated the effectiveness of natriuresis-guided diuretic therapy on natriuresis and clinical outcomes in patients with AHF.

## Results

**Fig. 1:**
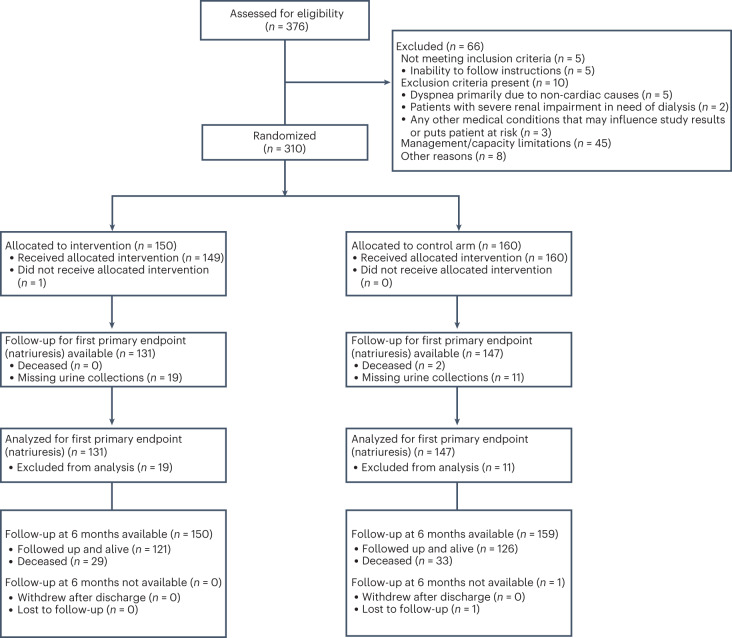
Patient flow diagram. Created with BioRender.com.

Between 11 February 2021 and 17 November 2022, we randomly assigned 310 patients to natriuresis-guided therapy (*n* = 150 (48.4%)) or the control group (*n* = 160 (51.6%)) (Fig. 1). The last patient completed follow-up on 9 May 2023. All patients provided informed consent following enrollment in the trial with deferred consent. The median age was 74 years [interquartile range 65–82 years], and 45% (*n* = 138) patients were female. The groups were similar in terms of baseline characteristics (Table 1). Patients in both groups had clinically important signs of congestion and a median N-terminal pro-blood natriuretic peptide (NT-proBNP) of 4,710 [2,553–8,750] ng l^−1^.

**Table 1:** Baseline clinical characteristics

	Natriuresis-guided therapy (*n* = 150)	SOC (*n* = 160)
**Demographics**		
Age (years)	74 [66–82]	74 [65–81.2]
Sex (% female (*n*))	41 (61)	48 (77)
Race (% white (*n*))	96 (142)	98 (155)
**Physical examination**		
Height (cm)	173 [166.2–180]	172 [165–179.5]
Weight (kg)	84 [71–92]	78 [70–93]
BMI (kg m^−2^)	27.3 [24.5–30.4]	28 [23.5–31.5]
Systolic blood pressure (mm Hg)	128 [110–150]	127.5 [113.5–147]
Diastolic blood pressure (mm Hg)	80 [68–92]	79 [69–94]
Heart rate (bpm)	89 [71–106]	94 [72–113]
Rales (% (*n*))	73 (108)	71 (109)
Ascites (% (*n*))	12 (12)	17 (16)
Edema (% (*n*))	68 (99)	67 (103)
Orthopnea (% (*n*))	67 (85)	72 (88)
NYHA class (% (*n*))		
II	3 (5)	7 (11)
III	26 (39)	18 (29)
IV	71 (106)	75 (120)
**HF**		
LVEF (%)	35 [25–53]	38 [28–48]
HFpEF (% (*n*))	26 (30)	18 (21)
Time since HF diagnosis (months)	7.5 [0–69.5]	14 [0–81.5]
New-onset HF (% (*n*))	44 (66)	43 (69)
Ischemic etiology (% (*n*))	37 (56)	34 (55)
**Medical history**		
History of atrial fibrillation (% (*n*))	56 (84)	56.2 (90)
History of diabetes (% (*n*))	34.7 (52)	41 (66)
History of hypertension (% (*n*))	62 (93)	64 (102)
**Laboratory values**		
Hemoglobin (mmol l^−1^)	7.7 [6.9–8.6]	7.8 [7–8.7]
Hematocrit (%)	0.4 [0.3–0.4]	0.4 [0.4–0.4]
Sodium (mmol l^−1^)	137 [133–140]	137 [134–140]
Potassium (mmol l^−1^)	4.3 [4.0–4.7]	4.3 [3.9–4.7]
Creatinine (µmol l^−1^)	106 [84–150]	106 [79–150]
Ureum (mmol l^−1^)	9.7 [6.3–14.8]	9.0 [6.4–14.4]
eGFR (ml min^−1^ 1.73 m^−2^)	54 [35–72]	53 [34.8–73.2]
NT-proBNP (ng l^−1^)	4,390 [2,554–8,226]	4,947 [2,607–9,809]
**Treatment**		
ACEi/ARB/ARNI (% (*n*))	55 (82)	54 (87)
Beta-blocker (% (*n*))	67 (100)	72 (115)
MRA (% (*n*))	35 (53)	34 (54)
SGLT2i (% (*n*))	5 (8)	8 (12)
Home loop diuretic (% (*n*))	56 (84)	58 (93)
Home loop diuretic dose (mg of bumetanide equivalents)	2 [1–4]	2 [1–4]
ICD (% (*n*))	22 (33)	22 (35)
CRT (% (*n*))	11 (17)	8 (12)

ACEi, angiotensin-converting enzyme inhibitor; ARB, angiotensin receptor blocker; ARNI, angiotensin receptor neprilysin inhibitor; BMI, body mass index; CRT, cardiac resynchronization therapy; HFpEF, HF with preserved ejection fraction; ICD, implantable cardiac defibrillator; MRA, mineralocorticoid receptor antagonist; NYHA class, New York Heart Association Class.

### Decongestive treatment

**Fig. 2:**
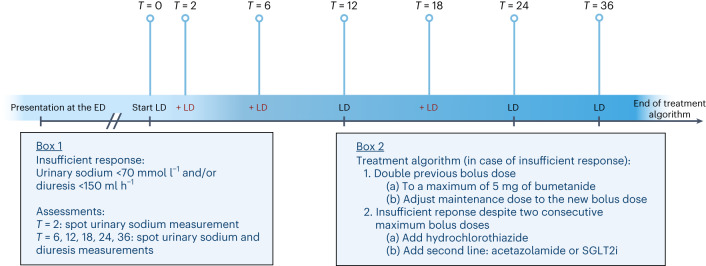
The urinary sodium and diuresis-based treatment protocol in PUSH-AHF. Schematic of the treatment protocol, showing that loop diuretics were administered twice daily (in black), at 12 h intervals. At the timepoints at which natriuresis and diuresis was assessed, in the case of an insufficient response as determined by predetermined cutoffs (box 1), treatment was intensified according to the treatment algorithm (box 2). First step was the administration of an additional dose of loop diuretics (double the previous dose to a maximum of 5 mg of bumetanide). If an additional, increased dose of loop diuretic was administered, the maintenance dose (the twice-daily administered loop diuretic dose) was further increased to a level that was double the previous dose, to a maximum of 5 mg bumetanide. If response continued to be insufficient despite two consecutive maximum doses of loop diuretic, combination diuretic therapy was started. First choice for combination diuretic therapy was the addition of hydrochlorothiazide; however, if a patient for instance already used combination diuretic therapy with hydrochlorothiazide before admission or response remained insufficient after addition of hydrochlorothiazide, acetazolamide or an SGLT2i was added. *T* refers to the time in hours after start of loop diuretic treatment. ED, emergency department; LD, loop diuretic. Created with BioRender.com.

**Extended Data Fig. 1:**
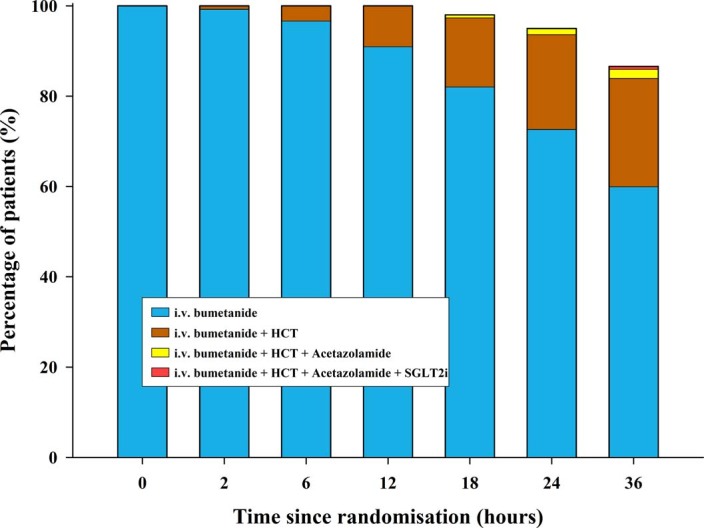
Intensification of treatment during the first 36 hours in the natriuresis guided group. *Abbreviations:* HCT: hydrochlorothiazide; SLGT2i: Sodium Glucose co-Transporter 2 inhibitor.

Median daily total dose of intravenous loop diuretic at the start of treatment was 4 [2–8] mg of bumetanide in the natriuresis-guided group compared with 4 [2–8] mg of bumetanide in the standard of care (SOC) group (*P* = 0.8082). The total cumulative administered inhospital diuretic dose was greater in the natriuresis-guided group (26 [15.5–44] mg of bumetanide) compared with the SOC group (15 [8.5–32] mg of bumetanide), *P* < 0.0001). In the natriuresis-guided group, in 128/150 individual patients (85%) diuretic treatment was intensified according to protocol (Extended Data Fig. 1) at any timepoint during the first 36 h (Fig. 2). Extended Data Table 1 displays the number of patients requiring treatment intensification at the different timepoints and the number of patients with an insufficient natriuretic response at these timepoints. In patients requiring treatment intensification, insufficient spot urinary sodium was the reason for intensified treatment in 100% (*n* = 28) of patients at *t* = 2, in 80% (*n* = 39) of patients at *t* = 6, in 63% (*n* = 47) of patients at *t* = 12, in 72% (*n* = 34) of patients at *t* = 18, and in 90% (*n* = 45) of patients at *t* = 36 (Extended Data Table 1). Response was most frequently insufficient 12 h after start of intravenous loop diuretic therapy (at *t* = 12), where 58% of patients in the natriuresis-guided arm had an insufficient response.

A cumulative total of 228 additional boluses of loop diuretics were administered in 123/150 patients in the natriuresis-guided group at all timepoints combined in the first 24 h. In 32/150 patients (21%) in the natriuresis-guided group a second diuretic was added (hydrochlorothiazide in 31 patients; acetazolamide in 3 patients). Two of these patients received triple nephron blockade with the addition of both hydrochlorothiazide and acetazolamide. In one patient in the natriuresis-guided group, a sodium glucose co-transporter 2 inhibitor (SGLT2i) was added according to protocol. No patients required ultrafiltration in the first 24 h of index hospitalization. In the first 24 h, the total administered loop diuretic dose in the natriuresis-guided arm was 12 [7–19] mg of bumetanide versus 6 [3–12] mg of bumetanide in the SOC arm (*P* < 0.0001).

At 36 h, 43 patients received an additional bolus of loop diuretic, in 6 patients a second diuretic was added (hydrochlorothiazide in 5 patients, and acetazolamide in 1 patient) and in 1 patient a SGLT2i was added per protocol. Of note, only 4/50 patients were eligible for treatment intensification at 36 h for the first time. In the first 36 h, the total administered loop diuretic dose in the natriuresis-guided arm was 16 [10–24] mg of bumetanide versus 8 [5–14] mg of bumetanide in the SOC arm (*P* < 0.0001).

**Extended Data Fig. 2:**
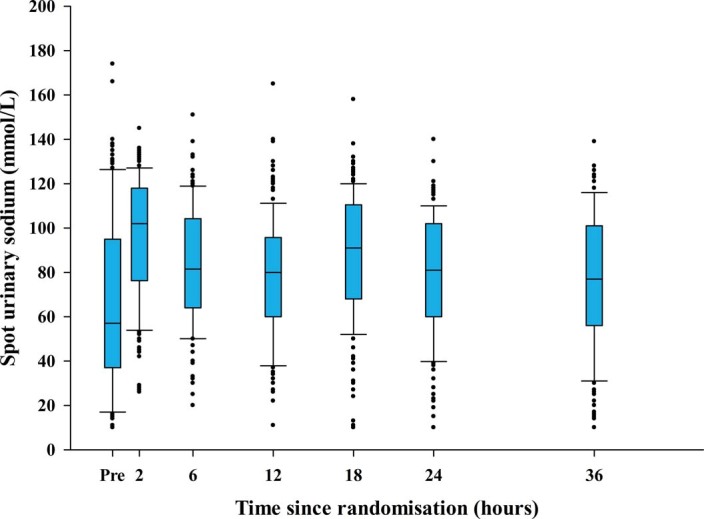
Spot urinary sodium values during the first 36 hours in the natriuresis guided group. Median with interquartile ranges, whiskers and outliers.

The median spot urinary sodium values in the natriuresis-guided arm during the first 36 h is shown in Extended Data Fig. 2. The median spot urinary sodium at baseline and at 2 h did not differ between the randomization groups (Extended Data Table 1).

### Primary endpoints

**Fig. 3:**
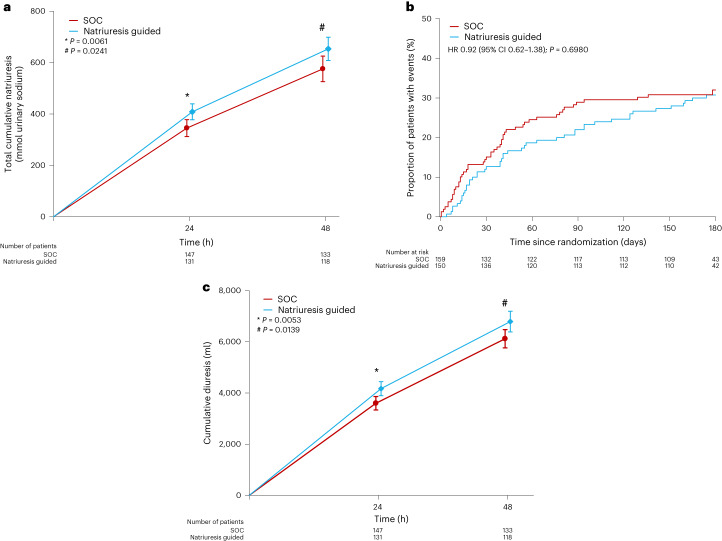
Natriuresis, diuresis and the combined endpoint of all-cause mortality and HF rehospitalization according to randomization group. **a**, Natriuresis at 24 and 48 h after start of loop diuretic (LD) treatment. Mean ± 95% CI. Student’s *t*-test. **b**, Kaplan–Meier plot for the combined primary endpoint of all-cause mortality and HF rehospitalization at 180 days. Cox regression. **c**, Diuresis at 24 and 48 h after start of LD treatment. Mean ± 95% CI. Student’s *t*-test.

**Extended Data Fig. 3:**
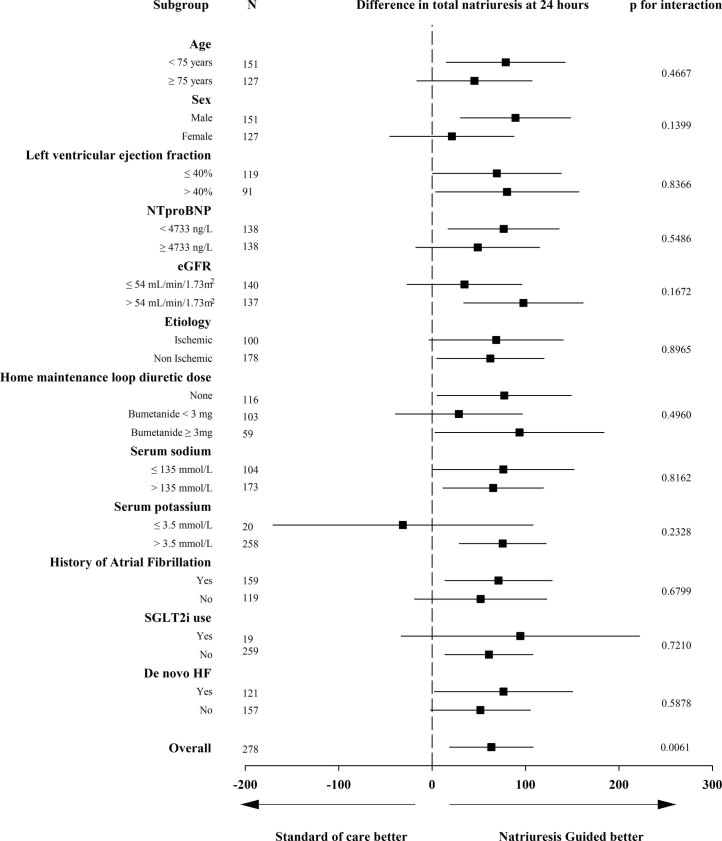
Subgroup analyses 24- hour natriuresis. Hazard ratio with 95% Confidence Intervals. *Abbreviations:* eGFR: estimated Glomerular Filtration Rate; HF: Heart Failure; NT-proBNP: N Terminal-pro Blood Natriuretic Peptide; SLGT2i: Sodium Glucose co-Transporter 2 inhibitor. *Linear regression analysis with the inclusion of an interaction term*.

Mean total 24 h natriuresis was 409 ± 178 mmol in the natriuresis-guided group compared with 345 ± 202 mmol in the SOC group (Table 2 and Fig. 3a) (*P* = 0.0061). The estimated difference in mean 24 h total natriuresis was 63 (95% confidence interval (CI) 18–109) mmol in favor of natriuresis-guided therapy. The effect of natriuresis-guided therapy on 24 h natriuresis was consistent across prespecified subgroups (Extended Data Fig. 3).

**Table 2:** Primary and secondary endpoints

	Natriuresis-guided therapy	SOC		*P* value
**Dual primary endpoint**			Estimated between-group difference (95% CI) or HR [95% CI] if applicable	
24 h natriuresis (mmol)^a^	409 ± 178	345 ± 202	63 (18–109)	0.0061
180 day HF rehospitalization or all-cause mortality (% (*n*))^b^	31 (46)	31 (50)	0.92 [0.62–1.38]	0.6980
**Secondary endpoints** (hierarchical testing)				
48 h natriuresis (mmol)	653 ± 249	575 ± 290	78 (10–145)	0.0241
24 h diuresis (ml)	3,900 [3,200–4,945]	3,330 (2,510–4,500)	534 (160–908)	0.0053
48 h diuresis (ml)	6,655 (5,401–7,824)	5,915 (4,600–7,400)	672 (137–1,206)	0.0140
Length of hospital stay (days)	6 [5–9]	7 [5–10]		0.1436
HF rehospitalization (% (*n*))	17 (25)	17 (26)	0.96 [0.56–1.67]	0.8904
Total number of HF rehospitalizations per patient	1 [1–1]	1 [1–1]		0.7663
180 day all-cause mortality (% (*n*))	19 (29)	21 (33)	0.89 [0.54–1.46]	0.6369
Percentage change in NT-proBNP (%)				
At 48 h	−22 [−48–12]	−18 [−41–17]		0.4351
At 72 h	−33 [−61–0]	−33 [−58–8]		0.7881

^a^The first part of the dual primary endpoint, 24 h total natriuresis, could not be assessed in 32 patients who underwent randomization (2 patients died within 24 h after admission, and 30 patients had missing urine collections). Student’s *t*-test.

^b^The second part of the dual primary endpoint, all-cause mortality or HF rehospitalization, could not be assessed in one patient who underwent randomization (lost to follow-up). Cox regression.

Normally distributed variables are tested with Student’s *t*-test, non-normally distributed values with Wilcoxon rank/sum test and categorical values with chi-square test.

**Extended Data Fig. 4:**
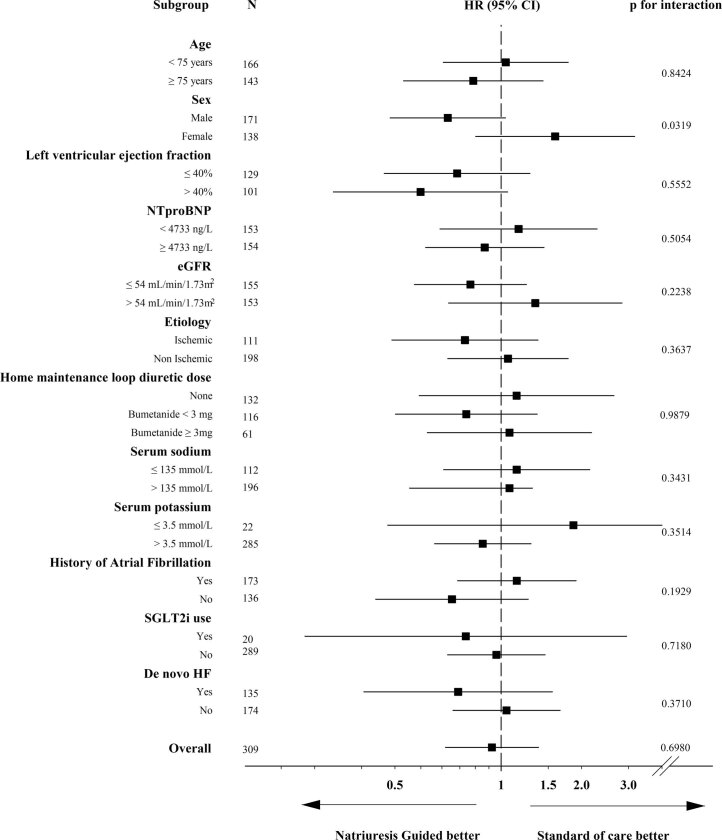
Subgroup analyses combined endpoint of all-cause mortality and heart failure rehospitalization at 180 days. Hazard ratio with 95% Confidence Intervals. *Abbreviations:* eGFR: estimated Glomerular Filtration Rate; HF: Heart Failure; NT-proBNP: N Terminal-pro Blood Natriuretic Peptide; SLGT2i: Sodium Glucose co-Transporter 2 inhibitor. *Cox regression analysis with the inclusion of an interaction term*.

All-cause mortality or HF rehospitalization at 180 days occurred in 46 of 150 patients (31%) in the natriuresis-guided group, and in 50 of 159 patients (31%) in the SOC group (hazard ratio (HR) 0.92 [95% CI 0.62–1.38], *P* = 0.6980) (Table 2 and Fig. 3b). The effect of natriuresis-guided therapy on all-cause mortality or HF hospitalization at 180 days was consistent across most prespecified subgroups (Extended Data Fig. 4).

A major protocol deviation occurred in 40 of 310 (14.2%) of all enrolled patients (28 (18.7%) in the natriuresis-guided group, and 16 (10.0%) in the SOC group). The per protocol analysis yielded similar findings (Extended Data Table 2).

### Secondary endpoints

**Extended Data Fig. 5:**
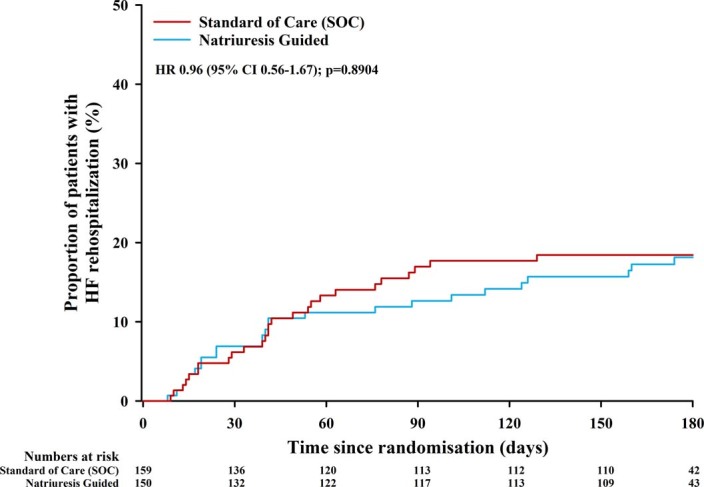
Kaplan Meier plot for heart failure rehospitalization at 180 days. *Abbreviations:* CI: Confidence Interval; HF: Heart Failure; HR: Hazard Ratio. *Cox regression*.

**Extended Data Fig. 6:**
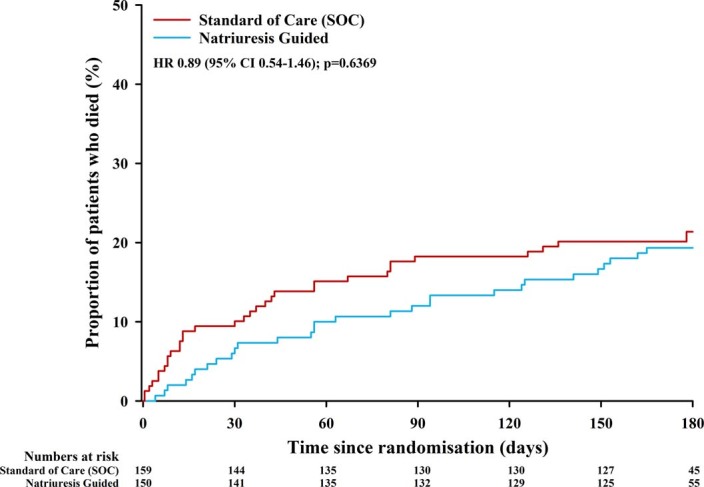
Kaplan Meier plot for all-cause mortality at 180 days. *Abbreviations:* CI: Confidence Interval; HR: Hazard Ratio. *Cox regression*.

In the natriuresis-guided group, mean 48 h natriuresis was 653 ± 249 mmol compared with 575 ± 290 mmol in the SOC group (Fig. 3a). The estimated difference in total natriuresis over 48 h was 78 (95% CI 10–145) mmol in favor of natriuresis-guided therapy (*P* = 0.0241). In the natriuresis-guided group, mean 24 h diuresis was 3,900 [3,200–4,945] ml compared with 3,330 [2,510–4,500] ml in the SOC group (Table 2, *P* = 0.0053). Forty-eight hour diuresis was also significantly greater in the natriuresis-guided therapy group compared with SOC (Table 2 and Fig. 3c). The duration of the index hospitalization was 6 [5–9] days in the natriuresis-guided group, compared with 7 [5–10] days in the SOC group (*P* = 0.8904). The incidence of HF hospitalizations and deaths as well as total number of HF hospitalizations per patient was not different between the randomized treatment groups in the natriuresis-guided group versus the SOC group (Table 2). There was also no difference in all-cause mortality or HF rehospitalization when analyzed as separate endpoints (Table 2 and Extended Data Figs. 5 and 6). Percentage change in NT-proBNP from baseline to 24 and 48 h was not different between the randomized treatment groups.

### Safety endpoints

Safety parameters during the index hospitalization were assessed in all patients who underwent randomization (Table 3). The predefined renal safety events (doubling of serum creatinine at 24 and 48 h) were scarce and did not differ between the randomization groups (0% in the natriuresis-guided group versus 1% in the SOC group). Additionally, no difference was found in the incidence of true worsening renal function (1% in the natriuresis group versus 1% in the SOC group). Worsening HF occurred in 9 (6%) patients in the natriuresis-guided group compared with 15 (9%) patients in the SOC group. The incidence of (serious) adverse events during 180 days of follow-up was similar in the two randomization groups (Table 3).

**Table 3:** Safety and exploratory endpoints

	Natriuresis-guided therapy	SOC	*P* value
**Safety endpoints**			
Serious adverse events (% (*n*))	40 (60)	44 (70)	0.5799
Adverse events (% (*n*))	57 (86)	60 (96)	0.7180
Renal safety events			
Doubling of serum creatinine at 24 h from baseline (% (*n*))	0 (0)	1 (1)	1.0000
Doubling of serum creatinine at 48 h from baseline (% (*n*))	1 (1)	1 (2)	1.0000
Worsening HF (% (*n*))	6 (9)	9 (15)	0.3689
True worsening renal function (% (*n*))	1 (1)	1 (2)	1.0000
**Exploratory endpoints**			
72 h natriuresis (mmol)	832 ± 323	746 ± 350	0.0706
72 h diuresis (ml)	8,720 [7,085–10,775]	8,255 [6,312.5–10,050]	0.1104
Fluid balance (ml)			
At 24 h	−2,833 ± 1,673	−2,380 ± 1,573	0.0218
At 48 h	−4,728 ± 2,318	−4,110 ± 2,137	0.0297
At 72 h	−6,216 ± 3,000	−5,728 ± 2,825	0.2400
Weight change from baseline (kg)			
At 24 h	−1.2 [−2.4–0.3]	−0.4 [−1.9–0.1]	0.1103
At 48 h	−3.5 [−5.0–1.6]	−3.2 [−4.4–1.0]	0.1505
At 72 h	−4.0 [−6.1–2.2]	−3.0 [−4.9–0.7]	0.1529
Percentage change in NT-proBNP (%)			
At 24 h	1 [−24–32]	3 [−15–26]	0.5103
Hypokalemia (K < 3.5 mmol l^−1^) (% (*n*))	23 (35)	15 (24)	0.0849
Hyperkalemia (K > 5.5 mmol l^−1^) (% (*n*))	2 (3)	6 (10)	0.1136

Normally distributed variables are tested with Student’s *t*-test, non-normally distributed values with Wilcoxon rank-sum test and categorical values with the chi-square test.

### Exploratory endpoints

**Extended Data Fig. 7:**
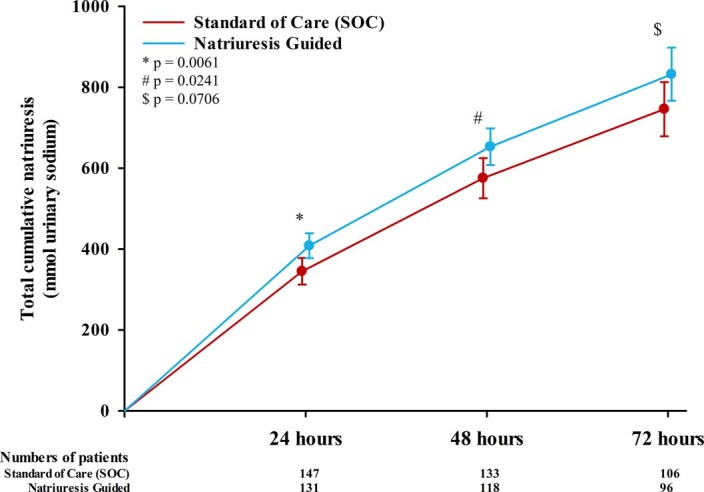
Natriuresis according to treatment group up to 72 hours. Mean ± 95% Confidence Interval. *Students T-test*.

**Extended Data Fig. 8:**
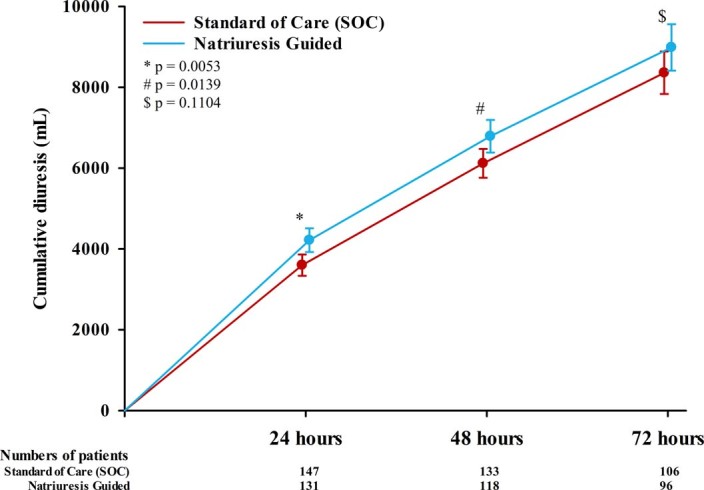
Diuresis according to treatment group up to 72 hours. Mean ± 95% Confidence Interval. *Students T-test*.

The difference in natriuresis and diuresis favoring the natriuresis-guided group was not sustained up to 72 h (Table 3 and Extended Data Figs. 7 and 8). Greater net fluid loss was obtained in the natriuresis-guided group versus the SOC group at 24 h; however, this became nonsignificant at the subsequent timepoints. There was no difference in weight loss or percentage change from baseline to 72 h between the randomized treatment groups. There was numerically more hypokalemia (defined as K < 3.5 mmol l^−1^) in the natriuresis-guided group (23%) compared with the SOC group (15%) (*P* = 0.0849).

## Discussion

In PUSH-AHF, natriuresis-guided diuretic therapy in patients with AHF significantly improved natriuresis and diuresis up to 48 h without impacting all-cause mortality and/or HF hospitalization at 180 days. There are several other key findings that deserve consideration in interpreting these results. First, in the natriuresis-guided treatment group, intensification of diuretic therapy occurred in the majority of patients, and this resulted in substantially higher doses of loop diuretics being administered. Second, the urinary sodium-based diuretic treatment algorithm was safe and did not result in renal or electrolyte perturbations despite much higher cumulative loop diuretic doses used. Third, the effect of natriuresis-guided therapy on 24 h natriuresis was consistent across a broad spectrum of patients reflecting a contemporary, all-comer, AHF population. The results of PUSH-AHF provide the first randomized evidence supporting the use of natriuresis-guided therapy to improve natriuresis in patients with AHF.

Over the past decades, diuretic treatment of AHF has been largely based on expert opinion and local practices using generally unreliable surrogate measures of response. Therefore, even nowadays, there is great variation in decongestive approaches and the use of diuretic therapy across centers and countries. This observation is even more important in the perspective of relatively recent data showing that impaired response to diuretics is common in patients with AHF and associated with persistent congestion and poor clinical outcomes, including high rates of HF rehospitalization^5–7^. There is an urgent and clinical need for a pragmatic, practical marker to assess response and to guide diuretic treatment to improve decongestion and subsequent clinical outcomes^12^.

To reduce congestion, loop diuretics are administered that inhibit the sodium-chloride–potassium co-transporter in the ascending loop of Henle resulting in potent natriuresis and subsequent diuresis^4,6^. HF is a sodium avid state, meaning that compensatory neurohormonal activation and renal adaptation is activated to retain as much sodium (and with it water) as possible^13^. Therefore, it could be argued that a net-negative sodium balance in patients presenting with congestion might even be a more important treatment goal. Even though greater natriuresis and a net negative balance has been associated with better outcomes, the question remains whether actively pursuing greater natriuresis is associated with better outcomes. Urinary sodium has the potential to not only serve as a diagnostic marker of diuretic response but may also hold potential as a guide for diuretic treatment. Furthermore, it is inexpensive, reliable and readily available using routine laboratory assessments. Because of this favorable biomarker profile, a combined natriuresis and diuresis-guided diuretic approach has already been incorporated in the most recent ESC HF guidelines^3^. However, so far, limited data to support this approach were available.

Following from the observation that early assessment of spot urinary sodium within 1–2 h after initiation of intravenous loop diuretic treatment was shown to be an accurate marker of subsequent 6 h natriuretic response, a natriuretic response prediction equation (NRPE) was developed^11^. In a prospective pre–post study, the implementation of the NRPE resulted in increased urine output, net fluid loss and weight loss compared with the days preceding this^14^. More recently, the nonrandomized results of the pre–post Effect of a Standardized Diuretic Protocol in Acute Heart Failure Study (ENACT) study were presented at the recent ESC HF congress (Protocolized natriuresis-guided decongestion improves diuretic response: the multicenter ENACT-HF study. Dauw, J. et al., submitted)^15^. Using a simplified version of the ESC HF guidelines treatment approach, natriuresis-guided loop diuretic therapy was associated with more natriuresis (282 mmol versus 174 mmol) at 24 h compared with patients treated with SOC. These findings of a significant effect of a natriuresis-guided therapy to increase natriuresis from ENACT are now confirmed with the randomized data from the PUSH-AHF trial.

The achieved natriuresis in the PUSH-AHF trial was much higher, also in the SOC group compared with previous observations from ENACT even in the active arm, and also from a subanalysis from the Acetazolamide in Decompensated Heart Failure with Volume Overload (ADVOR) study, where addition of acetazolamide resulted in total natriuresis of 258 ± 133 mmol in 24 h (refs. ^16,17^). These differences might be due to the inclusion of a different patient population where in the ADVOR trial patients were required to use loop diuretics at home, whereas the PUSH-AHF trial enrolled 44% of patients with de novo HF, frequently loop diuretic naïve with a higher likelihood of sufficient response to loop diuretic therapy. Additionally, in the PUSH-AHF trial higher doses of loop diuretics were administered during the trial compared with ADVOR and ENACT. Despite the observed higher achieved natriuresis in the SOC arm, intensification of loop diuretic treatment based on sequential spot urinary sodium values and diuresis was able to further increase natriuresis by a substantial amount. In parallel, as expected, diuresis was increased by a similar magnitude. It should be noted that a combined approach of insufficient spot urinary sodium and diuresis was employed where at all timepoints insufficient response was most frequently based on a spot urinary sodium <70 mmol. Similar to findings from ADVOR, the randomized treatment was mostly effective in the first 24 h of the intervention. For PUSH-AHF this was also the time during which monitoring and possible treatment alterations were most intense and frequent, although there was great variation in the timing of insufficient response based on spot sodium levels or diuresis. The effect of the intervention decreased after 48 h, possibly as a result of the end of the treatment algorithm after 36 h. Importantly despite the expected better response in loop diuretic naïve patients, there was no interaction for the effect of natriuresis-guided therapy on 24 h natriuresis in patients with new-onset versus established HF.

In contrast to the Diuretic Optimization Strategies Evaluation trial where the high-dose strategy was associated with greater diuresis but also with transient worsening of renal function, no increased risk of worsening of renal function was observed in the natriuresis-guided group^18^. This suggests that the individualized treatment approach based on insufficient natriuresis and diuresis, rather than prescribing high-dose loop diuretic therapy to all patients, identifies patients requiring additional decongestive therapy without the downside of renal function deterioration. Indeed, it has been shown that increased venous pressure as observed in congested patients with AHF is the strongest predictor of worsening renal function, and therefore treatment of congestion potentially has a renoprotective effect^19–21^. By specifically targeting patients requiring additional decongestive therapy based on insufficient natriuresis or diuresis a first step towards a personalized treatment approach of patients with AHF is taken. While the PUSH-AHF trial was pragmatic with regard to the design of the trial and the incorporation in the electronic health record (EHR), the implementation of the PUSH-AHF protocol in clinical practice might be considered less pragmatic given the frequency of assessments, also out-of-hours. However, incorporation of the PUSH-AHF protocol in the EHR could also facilitate clinical implementation as well as ultimately nurse-led execution of the treatment algorithm.

There was no effect of natriuresis-guided treatment on the combined endpoint of 180 day all-cause mortality and HF rehospitalization. It has been proven difficult to improve outpatient clinical outcome in patients with AHF, with the exception of some treatments that were continued after hospitalization. For PUSH-AHF, where the intervention was only administered during the first 36 h of admission, a direct effect may not be possible to observe. It would be tempting to speculate that there might have been an effect if the treatment algorithm was maintained until patients had reached euvolemia. There are currently multiple ongoing trials studying the effect of natriuresis-guided therapy that will provide additional data. A combined analysis with an ongoing randomized controlled trial studying the effect of protocolized diuretic therapy guided by spot urine chemistry using the NRPE to improve outcomes (ESCALATE; NCT04481919) is planned. Finally, given the neutral effect of natriuresis-guided therapy on the combined endpoint of 180 day all-cause mortality and HF rehospitalization, the predefined subgroup analyses should in our opinion be interpreted with caution.

We acknowledge the limitations of the single-center nature, as well as open-label design of this study. Given the design of the study, requiring adjustments based on urinary sodium levels, with our limited funding, it was unfortunately not feasible to perform this trial in a double-blind fashion including the pharmacist to blind the treating physicians to both the urinary sodium values as the necessity and dose of additional diuretics. We furthermore intentionally chose to design this study as a pragmatic trial to enroll a generalizable, all-comer, AHF population and allow for swift enrollment^12^. The additional incorporation in clinical care and the use of the EHR in the execution of this study all contributed to the pragmatic design of the trial and allowed us to perform this trial with limited funding. The pragmatic design also has some inherent drawbacks, such as no systematic assessment of congestion status, a larger number of missing data and the occurrence of protocol deviations. The PUSH-AHF illustrates that it is possible to successfully perform a trial investigating an early intervention in patients with AHF. The prespecified treatment algorithm was only maintained during the first 36 h of hospitalization possibly reducing the effect of the achieved increase in natriuresis during this time period. In the prespecified treatment algorithm, hydrochlorothiazide was added as first-choice second-line therapy. On the basis of the available data at the moment of study design, preceding the publication of the ADVOR trial, this was considered the best second-line option with most available evidence. Due to the single-center setting in the Netherlands, it remains unknown whether the findings of our trial are generalizable to non-white patients. Finally, we acknowledge that our study was probably underpowered to detect a smaller than hypothesized difference in clinical outcome, especially since the event rate was lower than anticipated.

In summary, the PUSH-AHF trial is the first randomized clinical trial to show that natriuresis-guided diuretic therapy improves natriuresis and diuresis in patients with AHF. These findings could be directly and easily implementable as spot urinary sodium values are easy to obtain, inexpensive and available in most centers around the world. Additionally, the studied treatment algorithm involves medication that are widely available, and is therefore easily implementable. An important observation from the PUSH-AHF trial is that natriuresis-guided diuretic therapy was safe and did not result in more (serious) adverse events or prespecified renal events. The PUSH-AHF study provides a first step towards a personalized natriuresis-guided approach in patients with AHF.

## Methods

### Study design

PUSH-AHF was a prospective, single-center, pragmatic, open-label, randomized, controlled clinical trial; the trial methods have been described previously^12^. The study was performed at the University Medical Center Groningen, the Netherlands, a tertiary hospital with an additional community function due to the limited number of community hospitals in our region. The trial protocol was approved by the ethics committee of the University Medical Center Groningen, the Netherlands (METC 2020/587), and is conducted in accordance with the Declaration of Helsinki and the International Conference of Harmonization Guidelines for Good Clinical Practice.

### Patients

The PUSH-AHF trial enrolled adult patients presenting with AHF requiring treatment with intravenous loop diuretics. Diagnosis of AHF was based on signs and symptoms, as indicated in the ESC HF guidelines, and could be either new onset or an exacerbation of known HF. The main exclusion criteria were severe renal impairment requiring ultrafiltration or dialysis, and dyspnea due to other causes. There was no ejection fraction or natriuretic peptide inclusion or exclusion criteria. The inclusion and exclusion criteria were left intentionally broad to enroll a contemporary, representative, all-comer AHF population. The full inclusion and exclusion criteria can be found in Supplementary Note 1. Further details on the design of the study have been reported previously^12^. The study protocol and statistical analysis plan are provided in Supplementary Note 2.

All participants provided written informed consent. The ethics committee of the University Medical Center Groningen approved the study for deferred consent on the basis of the necessity of swift enrollment at the emergency department and the low-risk nature of the study and intervention, hereby allowing immediate randomization after diagnosis and before start of treatment. Deferred (written) informed consent was obtained in all enrolled patients within the first 4 days of hospitalization.

### Randomization and masking

We aimed to randomly assign 50% of patients to either natriuresis-guided therapy or SOC. Randomization was done using the EHR (EPIC). To get random treatment allocation, a random number generator within the EHR was used that returned either 0 (zero) or 1. This variable was randomly generated within each individual patient file and hard-coded and could not be altered after the number had been generated. Via this way every patient had a 50% chance of being allocated to one of both randomized treatment groups—in analogy to ‘flipping a coin’. On the basis of this number, a study specific orderset was filled with treatment-arm-specific orders, which was ordered upon start of intravenous loop diuretic therapy. This trial was an open-label study. However, to prevent contamination and cross-over between treatment arms, physicians and investigators were blinded to all urinary sodium measurements (timed collections as well as spot urinary sodium) in the SOC arm. More details on the use of the EHR and blinding has been reported previously^12^.

### Procedures

In both treatment groups, baseline loop diuretic dose (the first inhospital dose of loop diuretics administered at the emergency department, irrespective of loop diuretic administration in the prehospital setting) was determined on the basis of the estimated glomerular filtration rate (eGFR) and outpatient loop diuretic dose (Supplementary Table 1). The maximum bolus dose was set at 5 mg of bumetanide. The bolus dose was consequently continued as twice daily dosing (every 12 h). In the SOC group, changes in diuretic dosing were not mandated by a set protocol, and left at the discretion of the treating physician. Per protocol, in the natriuresis-guided arm, spot urinary sodium samples were obtained at set timepoints (2, 6, 12, 18, 24 and 36 h). If urinary sodium values or diuresis (with the exception of 2 h) was insufficient, decongestive therapy was adjusted based on a prespecified treatment algorithm provided the patient was still congested (Fig. 2). A spot urinary sodium <70 mmol l^−1^ and/or diuresis <150 ml h^−1^ was considered insufficient. The prespecified treatment algorithm included an additional bolus of loop diuretic (double the previous bolus with a maximum dose of 5 mg of bumetanide). If a patient had received two doses of 5 mg of bumetanide at the two previous timepoints and had continued insufficient natriuresis or diuresis, the initiation of combination diuretic therapy was indicated. This included addition of 25 mg of hydrochlorothiazide, followed by acetazolamide (500 mg once daily)/SGLT2i and ultrafiltration as bail-out. After 48 h, adjustment of diuretic therapy was left at the discretion of the treating physician. More details are provided in Supplementary Figs. 1 and 2) and treatment protocol in Supplementary Note 2.

In both groups, 24 h urines were collected during the first 3 days of hospitalization. In the SOC group the values of these urine collections were blinded until the end of the study. Patients were contacted by telephone 180 days after enrollment to collect vital status and (serious) adverse events.

As defined in the statistical analysis plan, major protocol deviations were defined as those affecting the primary endpoint analyses (Supplementary Note 2).

### Outcomes

The dual primary endpoint was defined as (1) total 24 h natriuresis at day 1, and (2) time to first occurrence of all-cause mortality or HF rehospitalization until 180 days after randomization. Secondary endpoints were total 48 h natriuresis, total diuresis at 24 h, total diuresis at 48 h (0–48 h), length of hospital stay from baseline to discharge, time to first HF rehospitalization, number of HF rehospitalizations, time to death from any cause, number of deaths, and percentage change in NT-proBNP at 48 and 72 h. All rehospitalizations were adjudicated by the endpoint adjudication committee to judge whether a hospitalization was due to HF (Supplementary Note 1). The adjudication committee was blinded to the treatment allocation. Safety endpoints include serious adverse events, renal safety events and prespecified adverse events, including worsening HF during hospitalization and true worsening renal function (Supplementary Note 1). Renal safety events were defined as doubling of serum creatinine at 24 or 48 h from baseline. True worsening renal function was defined as doubling of serum creatinine from baseline to 48 or 72 h without evidence of decongestion, or urine production <10 cc h^−1^ despite adequate dosing of loop diuretics. Exploratory endpoints were total 72 h natriuresis, total diuresis at 72 h (0–72 h), net fluid balance at 24, 48 and 72 h, weight loss at 24, 48 and 72 h, percentage change in NT-proBNP at 72 h, and incidence of hypo- and hyperkalemia in the first 72 h.

### Statistical analysis

The sample size calculation is described in detail elsewhere^12^. A statistical power of 80% on mean change in total 24 h natriuresis at day 1 at a two-sided significance level of 0.025 (Bonferroni correction for the dual primary endpoint) was ensured if 125 patients per arm were available for this primary endpoint. For the second part of the dual primary endpoint (the combined endpoint of all-cause mortality or HF rehospitalization at 180 days), with 140 patients per group with available data, a statistical power of 81% again at a two-sided significance level of 0.025 was available to detect an HR of 0.49 with an anticipated event rate of 38% in the SOC arm.

Total 24 h natriuresis was normally distributed and calculated and presented as mean ± standard deviation. The between-group difference was tested using Student’s *t*-test. The effect of natriuresis-guided treatment on long-term outcomes was assessed using Cox regression (after checking proportional hazard assumption was met) for the between treatment difference. Kaplan–Meier estimates were calculated and plotted. For the presentation of baseline characteristics in both treatment arms, continuous variables are presented as mean ± standard deviation, non-normally distributed variables as median (25th–75th percentile) and categorical values as count (percentages).

We performed prespecified subgroup analyses of both components of the dual primary endpoint on the basis of the following covariates: age (≤/> median), sex, left ventricular ejection fraction (LVEF ≤40% versus >40%), NT-proBNP (≤/> median), eGFR (≤/> median), HF etiology (ischemic/non-ischemic), outpatient dose of loop diuretic use (yes/no)), hyponatremia (sodium ≤135 mmol l^−1^ versus >135 mmol l^−1^), hypokalemia (potassium ≤3.5 mmol l^−1^ versus >3.5 mmol l^−1^), atrial fibrillation (yes/no), SGLT2i (yes/no) and new-onset HF (yes/no). Kaplan–Meier estimates of the combined endpoint and its separate components by assigned treatment group were generated and presented as cumulative incidence curves.

All primary, secondary, safety and exploratory analyses were prespecified in the statistical analysis plan and performed in the intention-to-treat population (Supplementary Note 2). Primary and secondary endpoints were additionally assessed in the per protocol population. The statistical analysis plan did not include a coronavirus disease 2019 sensitivity analysis as this trial started after the coronavirus disease 2019 pandemic started in the Netherlands. The trial did not have a data safety monitoring board as it was considered a low-risk trial. Analyses were carried out using R version 4.0.2. For primary and secondary analyses, a two-tailed *P* value <0.025 was considered significant. For the safety and renal endpoints, a two-tailed *P* value <0.05 was considered significant. Given the open-label nature of the study, the primary endpoint analyses were performed by an independent statistician. Data were collected in REDCap, version 12.4.6. The trial was prospectively registered under the clinical trial registration number NCT04606927 at ClinicalTrials.gov.

### Role of the funding source

This study was funded by a personal Dutch Heart Foundation grant for J.M.t.M. (2020T012). The funder of the study had no role in study design, data collection, data analysis, data interpretation or writing of the manuscript.

### Reporting summary

Further information on research design is available in the Nature Portfolio Reporting Summary linked to this article.

## Online content

Any methods, additional references, Nature Portfolio reporting summaries, source data, extended data, supplementary information, acknowledgements, peer review information; details of author contributions and competing interests; and statements of data and code availability are available at 10.1038/s41591-023-02532-z.

### Supplementary information

Supplementary Information Supplementary Table 1, Figs. 1 and 2 and Notes 1 and 2.

Reporting Summary

## Figures and Tables

**Extended Data Table 1:**
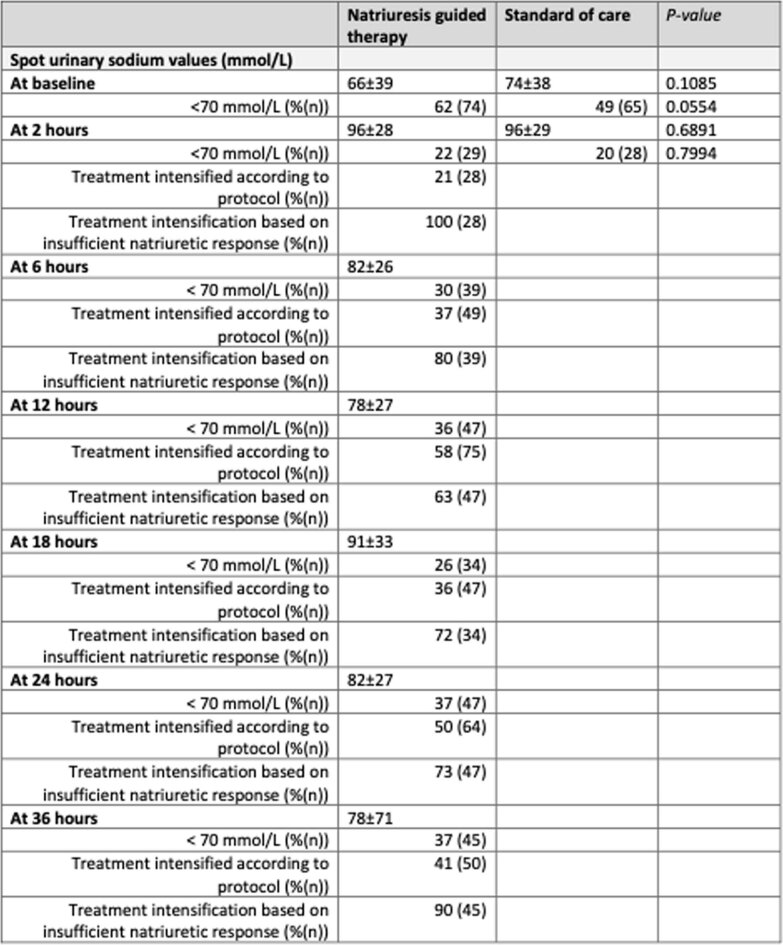
Spot urinary sodium values during the first 36 hours of hospitalization

Students T-test.

**Extended Data Table 2:**
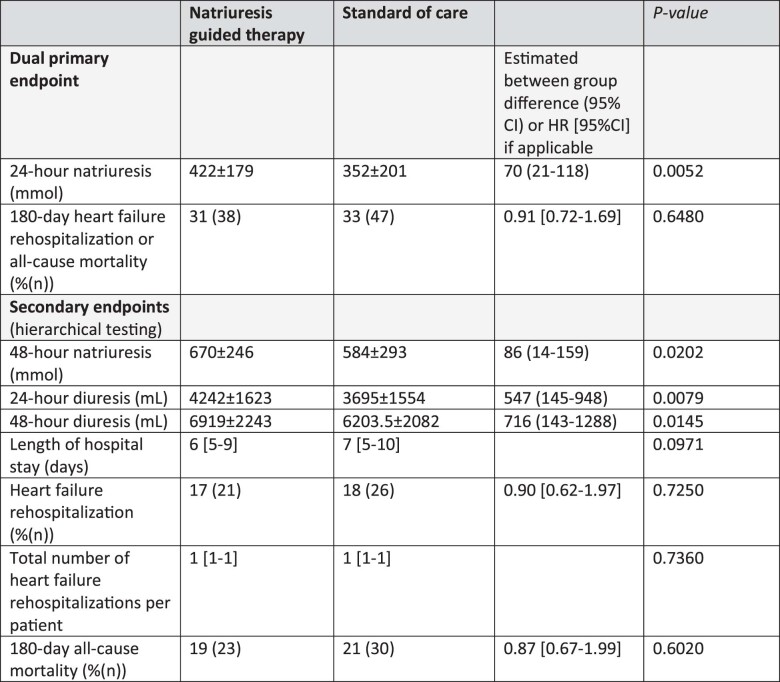
Per protocol analyses of primary and secondary endpoints

Normally distributed variables are tested with Students T-test, non-normally distributed values with Wilcoxon rank sum test, and categorical values with Chi square test. The time dependent outcome analysis is tested using Cox regression.

## Data Availability

Anonymized participant data can be made available upon requests directed to the corresponding author. Proposals will be reviewed on the basis of scientific merit, ethical review, available resources and regulatory requirements. After approval of a proposal, anonymized data will be made available for reuse. A steering committee will have the right to review and comment on any draft papers based on these data before publication.

## References

[1] Gheorghiade M, Vaduganathan M, Fonarow GC, Bonow RO (2013). Rehospitalization for heart failure: problems and perspectives. J. Am. Coll. Cardiol..

[2] Groenewegen A, Rutten FH, Mosterd A, Hoes AW (2020). Epidemiology of heart failure. Eur. J. Heart Fail..

[3] McDonagh TA (2021). 2021 ESC Guidelines for the diagnosis and treatment of acute and chronic heart failure. Eur. Heart J..

[4] Mullens W (2019). The use of diuretics in heart failure with congestion—a position statement from the Heart Failure Association of the European Society of Cardiology. Eur. J. Heart Fail..

[5] Valente MAE (2014). Diuretic response in acute heart failure: clinical characteristics and prognostic significance. Eur. Heart J..

[6] ter Maaten JM (2015). Diuretic response in acute heart failure—pathophysiology, evaluation, and therapy. Nat. Rev. Cardiol..

[7] Testani JM (2014). Loop diuretic efficiency: a metric of diuretic responsiveness with prognostic importance in acute decompensated heart failure. Circ. Heart Fail..

[8] Singh D (2014). Insufficient natriuretic response to continuous intravenous furosemide is associated with poor long-term outcomes in acute decompensated heart failure. J. Card. Fail..

[9] Hodson, D. Z. et al. Natriuretic response is highly variable and associated with 6-month survival: insights from the ROSE-AHF trial. *JACC Heart Fail.* **7**, 383–391 (2019). 10.1016/j.jchf.2019.01.007 PMC6501816 31047017

[10] Damman K (2020). Clinical importance of urinary sodium excretion in acute heart failure. Eur. J. Heart Fail..

[11] Testani JM (2016). Rapid and highly accurate prediction of poor loop diuretic natriuretic response in patients with heart failure. Circ. Heart Fail..

[12] Ter Maaten JM (2022). Natriuresis-guided therapy in acute heart failure: rationale and design of the Pragmatic Urinary Sodium-based treatment algoritHm in Acute Heart Failure (PUSH-AHF) trial. Eur. J. Heart Fail..

[13] Mullens W, Verbrugge FH, Nijst P, Tang WHW (2017). Renal sodium avidity in heart failure: from pathophysiology to treatment strategies. Eur. Heart J..

[14] Rao VS (2021). Natriuretic equation to predict loop diuretic response in patients with heart failure. J. Am. Coll. Cardiol..

[15] Dauw J (2021). Rationale and design of the efficacy of a standardized diuretic protocol in acute heart failure study. Esc. Heart Fail..

[16] Verbrugge FH (2023). Natriuretic response to acetazolamide in patients with acute heart failure and volume overload. J. Am. Coll. Cardiol..

[17] Mullens W (2022). Acetazolamide in acute decompensated heart failure with volume overload. N. Engl. J. Med..

[18] Felker GM (2011). Diuretic strategies in patients with acute decompensated heart failure. N. Engl. J. Med..

[19] Mullens W (2008). Elevated intra-abdominal pressure in acute decompensated heart failure: a potential contributor to worsening renal function?. J. Am. Coll. Cardiol..

[20] Mullens W (2009). Importance of venous congestion for worsening of renal function in advanced decompensated heart failure. J. Am. Coll. Cardiol..

[21] Damman K (2009). Increased central venous pressure is associated with impaired renal function and mortality in a broad spectrum of patients with cardiovascular disease. J. Am. Coll. Cardiol..

