# Supplementary material for: Natriuresis-guided diuretic therapy in acute heart failure: a pragmatic randomized trial
Source: Nat Med. 2023 Aug 28;29(10):2625–32. doi: 10.1038/s41591-023-02532-z (PMC10579092; doi:10.1038/s41591-023-02532-z)
Supplement: Supplementary file 1 — Supplementary Table 1, Figs. 1 and 2 and Notes 1 and 2. [file 41591_2023_2532_MOESM1_ESM.pdf]

nature medicine

Article https://doi.org/10.1038/s41591-023-02532-z

Natriuresis-guided diuretic therapy in acute heart failure: a pragmatic randomized trial

In the format provided by the authors and unedited

Supplementary table S1. Starting dose of loop diuretics (in all patients):

	Loop diuretic naive	Chronic loop diuretic use
eGFR ≥60 ml/min/1.73m^2	Bolus of 1 mg of bumetanide	Bolus equal to total daily loop diuretic dose at home
eGFR <60 ml/min/1.73m^2	Bolus of 2 mg of bumetanide	Bolus double the total daily loop diuretic dose at home
Maintenance dose is twice daily bolus dose		

* 40 mg of furosemide is considered equal to 1 mg of bumetanide

* Maximum bolus dose is 5 mg of bumetanide

Table legend: eGFR: estimated Glomerular Filtration Rate

Supplementary figure S1. Treatment protocol in the natriuresis guided arm during the first 24 hours (0-24 hours after randomization)

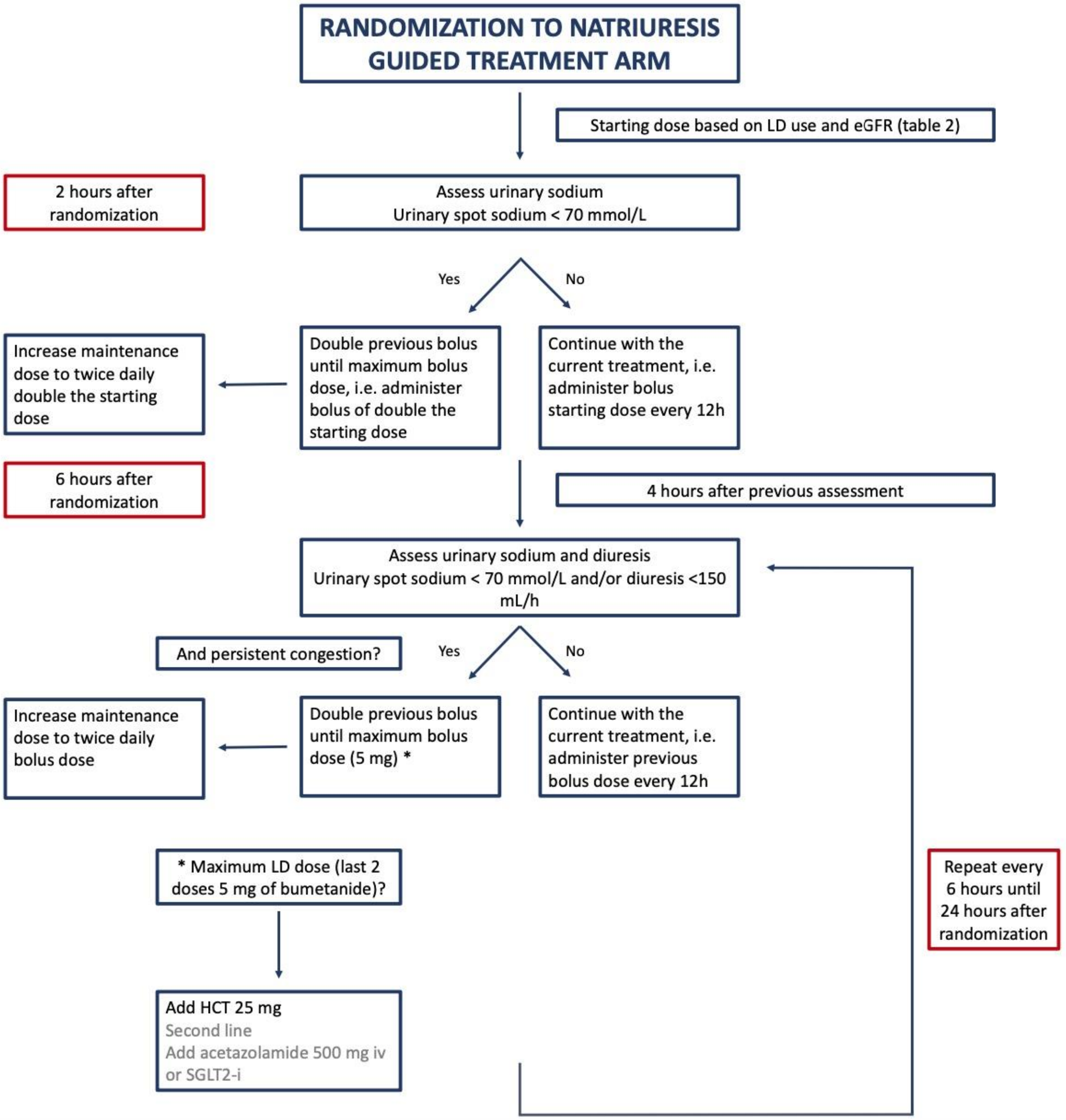

Abbreviations: eGFR: estimated Glomerular Filtration Rate, HCT: hydrochlorothiazide, LD: loop diuretic, SGLT2-i: sodium-glucose co-transporter inhibitor

Supplementary figure S2. Treatment protocol in the natriuresis guided arm during the second 24 hours (24-48 hours after randomization)

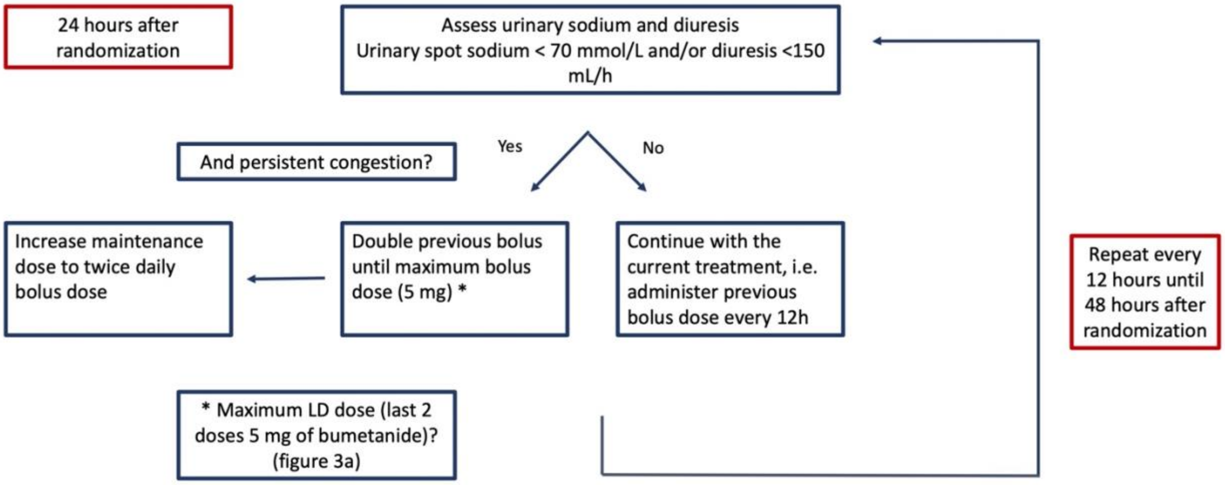

Abbreviation: LD: loop diuretic

PUSH-AHF

Natriuresis guided diuretic therapy in acute heart failure (PUSH-AHF): a randomized clinical trial

Supplementary note 1

Table of contents

P. 2 PUSH-AHF inclusion / exclusion criteria

P. 3 Endpoint (HF rehospitalization) definition

P. 4 Prespecified adverse event definitions

PUSH AHF inclusion / exclusion criteria

<u>Inclusion criteria:</u>

- Male or female ≥18 years of age
- Primary diagnosis of acute/decompensated heart failure as assessed by treating physician
  - Acute heart failure can be either *de novo* or an exacerbation of known heart failure
  - Diagnosis is based on criteria in the ESC HF guidelines
- Requirement of intravenous loop diuretic use

<u>Exclusion criteria:</u>

- Dyspnea primary due to non-cardiac causes
- Patients with severe renal impairment receiving dialysis or requiring ultrafiltration
- Inability to follow instructions
- Previous participation in this study
- Any other medical conditions that may put the patient at risk or influence study results in the investigator's opinion, or that the investigator deems unsuitable for the study

Endpoint (HF rehospitalization) definition

A hospitalization for heart failure will be defined as follows:
Hospitalization for more than one day (change in calendar day) with an exacerbation of heart failure requiring treatment meeting the following criteria:

- Signs and symptoms of heart failure
    - One or more of the following symptoms consistent with heart failure:
        - Dyspnea
        - Orthopnea
        - Paroxysmal nocturnal dyspnea
        - Increasing fatigue/decreasing exercise tolerance
        - Edema/anasarca
        - Other symptoms of worsened end-organ perfusion such as dizziness, mental confusion
    - AND two or more of the following sign consistent with heart failure:
        - Weight gain
        - Pulmonary edema or rales
        - Elevated jugular venous pressure
        - Radiologic signs of heart failure
        - Peripheral edema
        - Abdominal distension or ascites
        - S3 gallop rhythm
        - Positive hepatojugular reflux
        - Elevated NT-proBNP
        - Congestive hepatomegaly
        - Invasive/non-invasive tests showing elevated cardiac filling pressures or low cardiac output
- AND treatment
    - The patient receives initiation or intensification of treatment specifically for heart failure

Prespecified adverse event definitions

Pre-specified adverse events are defined as follows:

- Worsening heart failure defined as:
  - Addition or starting of inotropes or vasopressors
  - Mechanical ventilation
  - Palliative care due to progressive heart failure
  - Any intervention/treatment for heart failure that leads to prolonged hospitalization
- True worsening renal function
  - Doubling of creatinine from baseline to 48 or 72 hours without evidence of decongestion, or urine production < 10 cc / hour despite adequate dosing of loop diuretics

PUSH-AHF

Natriuresis guided diuretic therapy in acute heart failure (PUSH-AHF): a randomized clinical trial

Supplementary note 2

Table of contents

P. 2 PUSH-AHF clinical trial protocol

P. 45 Statistical Analysis Plan

Protocol Investigator Initiated Study

PUSH AHF

Pragmatic Urinary Sodium-based treatment algoritHm in Acute Heart Failure

Version number: 05, 17 October 2021

PROTOCOL TITLE 'Pragmatic urinary sodium-based treatment algorithm in acute heart failure'

**Protocol ID**	**PUSH AHF**
**Short title**	**Urinary sodium based treatment in AHF**
**EudraCT number**	**NA**
**Version**	**4.0**
**Date**	**17 October 2021**
**Coordinating investigator/project leader**	**J.M. ter Maaten, MD, PhD**<br>**University Medical Center Groningen**<br>**Department of Cardiology**<br>**P.O. Box 30 001, 9700 RB Groningen**<br>**R. +31 (0) 50 361 2355**<br>**Email: j.m.ter.maaten@umcg.nl**
**Principal investigator(s) (in Dutch: hoofdonderzoeker/ uitvoerder)**	**J.M. ter Maaten, MD, PhD**<br>**University Medical Center Groningen**<br>**Department of Cardiology**<br>**P.O. Box 30 001, 9700 RB Groningen**<br>**R. +31 (0) 50 361 2355**<br>**Email: j.m.ter.maaten@umcg.nl**<br>**K. Damman, MD, PhD**<br>**University Medical Center Groningen**<br>**Department of Cardiology**<br>**P.O. Box 30 001, 9700 RB Groningen**<br>**R. +31 (0) 50 361 2355**<br>**Email: k.damman@umcg.nl**
**Sponsor (in Dutch: verrichter/opdrachtgever)**	**Investigator initiated study**

**Subsidising party**	**Hartstichting**
**Independent expert (s)**	**Dr. P.P. van Geel**
**Laboratory sites**	**NA**
**Pharmacy**	**NA**

PROTOCOL SIGNATURE SHEET

Name	Signature	Date
Sponsor or legal representative:<br>Head of Department of Cardiology:<br>Prof. dr. D.J. van Veldhuisen	[signature]	18-10-'21
Coordinating Investigator:<br>Dr. J.M. ter Maaten	[signature]	17-10-'21

TABLE OF CONTENTS

1. INTRODUCTION AND RATIONALE 9
2. OBJECTIVES 10
3. STUDY DESIGN 11
3.1 Study visits 11
3.2 Screening 15
3.3 Assessment 1 (Baseline) 15
3.4 Assessment 2 (2 hours) 15
3.5 Assessment 3.0 (6 hours) 16
3.6 Assessment 3.1 (12 hours) 16
3.7 Assessment 3.2 (18 hours) 16
3.8 Assessment 4.0 (24 hours) 17
3.9 Assessment 4.1 (36 hours) 17
3.10 Assessment 5 (48 hours) 17
3.11 Assessment 6 (72 hours) 18
3.12 Assessment 7 (discharge) 18
3.13 Assessment 8 (180 days +/- 10 days) 19
3.14 Treatment algorithm 19
3.14.1 Loop diuretic dose at baseline 20
3.14.2 Standard of care group 21
3.14.3 Natriuresis determined treatment algorithm 22
3.15 Renal ultrasound substudy 24
3.16 Pharmacodynamic substudy 26
4. STUDY POPULATION 27
4.1 Population (base) 27
The study population consist of male and female patients (≥18 years old) admitted to the hospital for acute heart failure and requiring intravenous diuretic therapy at the University Medical Centre Groningen. 27
4.2 Inclusion criteria 27
4.3 Exclusion criteria 27
4.4 Sample size calculation 27
5. TREATMENT OF SUBJECTS 29
5.1 Investigational product/treatment 29
5.2 Use of co-intervention (if applicable) 29
5.3 Escape medication (if applicable) 29
6. METHODS 30
6.1 Study parameters/endpoints 30
6.1.1 Main study parameter/endpoint 30
6.1.2 Secondary study parameters/endpoints (if applicable) 30
6.1.3 Other study parameters (if applicable) 30
6.2 Randomization, blinding and treatment allocation 30
6.3 Study procedures 31

6.4 Withdrawal of individual subjects 32
6.4.1 Specific criteria for withdrawal (if applicable) 32
6.5 Replacement of individual subjects after withdrawal 32
6.6 Follow-up of subjects withdrawn from treatment 32
6.7 Premature termination of the study 32
7. SAFETY REPORTING 33
7.1 Temporary halt for reasons of subject safety 33
7.2 AEs, SAEs and SUSARs 33
7.2.1 Adverse events (AEs) 33
7.2.2 Serious adverse events (SAEs) 33
7.2.3 Suspected unexpected serious adverse reactions (SUSARs) 34
7.3 Annual safety report 34
7.4 Follow-up of adverse events 34
7.5 Endpoint adjudication committee 34
STATISTICAL ANALYSIS 36
7.6 Primary study parameter(s) 36
7.7 Secondary study parameter(s) 36
7.8 Other study parameters 36
7.9 Interim analysis (if applicable) 36
Not applicable 36
8. ETHICAL CONSIDERATIONS 37
8.1 Regulation statement 37
8.2 Recruitment and consent 37
8.3 Objection by minors or incapacitated subjects (if applicable) 37
8.4 Benefits and risks assessment, group relatedness 37
8.5 Compensation for injury 38
8.6 Incentives 38
9. ADMINISTRATIVE ASPECTS, MONITORING AND PUBLICATION 39
9.1 Handling and storage of data and documents 39
9.2 Monitoring and Quality Assurance 39
9.3 Amendments 39
9.4 Annual progress report 39
9.5 Temporary halt and (prematurely) end of study report 39
9.6 Public disclosure and publication policy 40
unreservedly. 40
10. STRUCTURED RISK ANALYSIS 41
10.1 Potential issues of concern 41
10.2 Synthesis 41
11. REFERENCES 42

LIST OF ABBREVIATIONS AND RELEVANT DEFINITIONS

**AE**	**Adverse Event**
**AHF**	**Acute Heart Failure**
**ECG**	**Electrocardiogram**
**eGFR**	**Estimated Glomerular Filtration Rate**
**EHR**	**Electronic Health Record**
**ESC**	**European Society of Cardiology**
**EudraCT**	**European drug regulatory affairs Clinical Trials**
**GCP**	**Good Clinical Practice**
**HCT**	**Hydrochlorothiazide**
**HF**	**Heart Failure**
**IC**	**Informed Consent**
**JVP**	**Jugular Venous Pressure**
**LD**	**Loop Diuretic**
**METC**	**Medical research ethics committee (MREC); in Dutch: medisch ethische toetsing commissie (METC)**
**NT-proBNP**	**N-terminal pro blood natriuretic peptide**
**NYHA**	**New York Heart Association**
**(S)AE**	**(Serious) Adverse Event**
**SGLT2i**	**Sodium Glucose Transporter 2 inhibitor**
**SOC**	**Standard Of Care**
**Sponsor**	**The sponsor is the party that commissions the organisation or performance of the research, for example a pharmaceutical company, academic hospital, scientific organisation or investigator. A party that provides funding for a study but does not commission it is not regarded as the sponsor, but referred to as a subsidising party.**
**SUSAR**	**Suspected Unexpected Serious Adverse Reaction**
**UMCG**	**University Medical Centre Groningen**
**VAS**	**Visual Analog Scale**
**WMO**	**Medical Research Involving Human Subjects Act (in Dutch: Wet Medisch-wetenschappelijk Onderzoek met Mensen**

SUMMARY

Rationale: Administration of loop diuretics to achieve decongestion is the current cornerstone of therapy for acute heart failure. Unfortunately, there is a lack of evidence of how to guide diuretic treatment. Recently, urinary sodium, as a response measure of diuretic response, has been proposed as a target for therapy.

The hypothesis of this study is that natriuresis guided therapy in patients with acute heart failure will improve diuretic response, decongestion, and reduce length of hospital stay, as well as heart failure rehospitalisations.

Objective: To assess the effect of natriuresis guided therapy in acute heart failure to improve diuretic response, decongestion, and clinical outcomes

Study design: Randomised, controlled, open label study

Study population: 310 patients admitted with the primary diagnosis of acute heart failure requiring intravenous loop diuretics.

Intervention (if applicable): natriuresis guided treatment versus standard of care

Main study parameters/endpoints:

Co-primary outcome: total natriuresis after 24 hours and first occurrence of all-cause mortality or heart failure rehospitalisation at 6 months

Secondary outcomes: 48- and 72-hours natriuresis, length of hospital stay, percentage change in NT-proBNP at 48 and 72 hours.

Safety endpoints: doubling of serum creatinine at 24 or 48 hours, occurrence of worsening heart failure

Nature and extent of the burden and risks associated with participation, benefit and group relatedness: Since this is a pragmatic trial, the study will be embedded within the normal care of patients with acute heart failure, which already includes timed urinary collections and laboratory assessment at set time points. The patients in the natriuresis guided therapy will undergo additional urinary assessments, and undergo more stringent monitoring of response, and therefore might receive more intravenous diuretics. For study parameters, blood and urine will be collected at set time points. Survival and rehospitalisation will be assessed after 6 months by telephone call.

1. INTRODUCTION AND RATIONALE

Heart failure is one of the leading causes of hospitalization in the world, is associated with significant morbidity and mortality, and as such responsible for a large proportion of health care expenses.(1) It is estimated that the total number of heart failure patients will continue to increase. An increase of 88% in 2040 is predicted, resulting in a further increase of health care expenditures attributable to heart failure.(1) While treatment for chronic heart failure has improved greatly over the last decades, this is not true for acute heart failure (AHF) where therapies with a proven positive effect on outcome are non-existent. Additionally, a large number of AHF patients show impaired response to the only available therapy, i.e. loop diuretics.(2) Over the last years several studies consistently showed that impaired diuretic response is associated with residual congestion and an increased risk of mortality and heart failure rehospitalisations.(3-7) Given the working mechanisms of loop diuretics, natriuresis might be a more sensitive, objective, quantifiable, and reliable marker to assess response. We recently showed that insufficient natriuretic response in AHF patients was indeed associated with an increased risk of poor outcome.(8) Additionally, even early assessment of natriuresis (one to two hours after initiation of loop diuretics) in AHF patients has been shown to be an accurate marker of insufficient diuretic response during hospitalization.(9) Therefore, natriuresis might have a role in actively assessing response to loop diuretics and to subsequently guide diuretic treatment in AHF patients. Natriuresis possesses all the characteristics required for a marker that can be used to actively guide decongestive treatment and move towards a personalized treatment approach in AHF. Interventions aimed at improving diuretic response, using natriuresis guided therapy have the potential to significantly improve effectiveness of decongestion, speed up inhospital treatment, prevent readmissions for heart failure, and decrease health care expenses (figure 1). We therefore hypothesize that *natriuresis guided therapy* in patients with AHF will *improve diuretic response, decongestion*, and *reduce length of hospital stay*, as well as *heart failure rehospitalisations.*

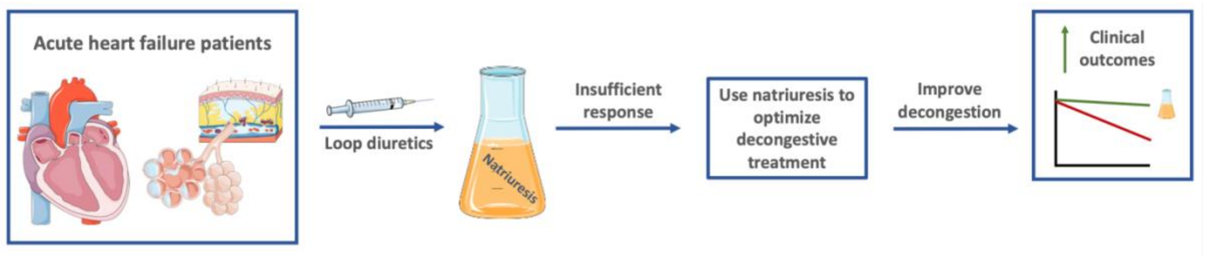

Figure 1.

Legend: in patients with AHF, loop diuretics are the first and only recommended choice of treatment aimed at relieving congestion by increasing diuresis and natriuresis. Actively assessing natriuresis and using this to optimize diuretic treatment could improve decongestion and clinical outcomes.

2. OBJECTIVES

Primary Objective:

To assess the effect of natriuresis guided therapy versus standard of care (SOC) on 24-hour urinary sodium excretion after the start of intravenous loop diuretic and the effect on first occurrence of all-cause mortality or heart failure rehospitalisation at 6 months in patients with acute heart failure

Secondary Objective(s):

To assess the effect of natriuresis guided therapy on:

- 48- and 72 hours urinary sodium excretion
- Length of hospital stay
- Percentage change in NT-proBNP at 48 and 72 hours

Safety endpoints:

- Doubling of serum creatinine at 24 or 48 hours
- Occurrence of worsening heart failure

3. STUDY DESIGN

This is a pragmatic, randomized, controlled, open label study in patients presenting with acute heart failure requiring intravenous loop diuretics. In total 310 patients will be randomized at the start of intravenous treatment (inhospital) to standard of care or natriuresis guided treatment. Due to the severe illness of acute heart failure patients, and the necessity to perform the first study assessment early (i.e. within the first hours of hospitalization) patients will be enrolled at the emergency department using deferred consent. Written informed will be obtained during the first four days of hospitalization. Patients will be followed for the duration of the hospitalization. Adverse clinical events, including all-cause mortality and heart failure rehospitalisation will be assessed by a telephone call after 6 months.

3.1 Study visits

Table 1 lists all of the assessments and indicates with an “X” when the required assessments and/or procedures are performed at a scheduled visit day. All laboratory assessments, including blood and urine assessment for the treatment algorithm and for the primary outcome will be integrated in clinical practice. The results of the urinary assessments will be blinded in the standard of care group during the study duration.

Study candidates will be identified upon arrival at the emergency department. When a patient is diagnosed with AHF and intravenous diuretic therapy is started, randomization will occur using Epic (Epic Systems Corporation). The study procedures will then start according to this protocol. Patients will receive a urinary catheter if this is deemed necessary by the treating physician.

Table 3.1 Assessment schedule

	**Screening**	**Participation**										**Follow-up**
**Time points**	Hour -6	Hour 0 (day 1)	Hour 2 (day 1) [d]	Hour 6 (day 1)	Hour 12 (day 1) [d]	Hour 18 (day 1) [d]	Hour 24 (day 1)	Hour 36 (day 2) [d]	Hour 48 (day 2)	Hour 72 (day 3)	Discharge	Day 180 (+/- 10 days) [a]
**Visits**	0	1	2	3.0	3.1	3.2	4.0	4.1	5	6	7	8
Written informed consent							X		X	X		
Inclusion/exclusion criteria	X											
Demography	X											
Medical history	X											
Heart failure history	X											
Concomitant medication	X	X					X		X	X	X	X
Physical examination	X	X		X			X		X	X	X	
Height	X											
Weight	X	X					X		X	X	X	X

	**Screening**	**Participation**										**Follow-up**
**Time points**	Hour -6	Hour 0 (day 1)	Hour 2 (day 1) [d]	Hour 6 (day 1)	Hour 12 (day 1) [d]	Hour 18 (day 1) [d]	Hour 24 (day 1)	Hour 36 (day 2) [d]	Hour 48 (day 2)	Hour 72 (day 3)	Discharge	Day 180 (+/- 10 days) [a]
**Visits**	0	1	2	3.0	3.1	3.2	4.0	4.1	5	6	7	8
Blood pressure and heart rate measurements	X	X	X	X	X	X	X	X	X	X	X	
NYHA class	X	X					X		X	X	X	X
Physician assessment of HF signs and symptoms	X	X		X			X		X	X	X	
Echocardiogram [b]												
X-ray [c]	X											
ECG	X											
Loop diuretics	X	X	X	X	X	X	X	X	X	X	X	X
Standard local laboratory analysis	X			X			X		X	X	X	
Timed urine collection (24h)							X		X	X		

	Screening	Participation										Follow-up
**Time points**	Hour -6	Hour 0 (day 1)	Hour 2 (day 1) [d]	Hour 6 (day 1)	Hour 12 (day 1) [d]	Hour 18 (day 1) [d]	Hour 24 (day 1)	Hour 36 (day 2) [d]	Hour 48 (day 2)	Hour 72 (day 3)	Discharge	Day 180 (+/- 10 days) [a]
**Visits**	0	1	2	3.0	3.1	3.2	4.0	4.1	5	6	7	8
Spot natriuresis		X	X	X	X	X	X	X	X	X	X	
Urine sample		X	X	X			X		X	X	X	
Plasma sample		X		X			X		X	X	X	
Fluid intake assessment							X		X	X		
Diuresis assessment			X	X	X	X	X	X	X	X		
Vital status and outcome assessments		X					X		X	X	X	X
Events and serious adverse events		X					X		X	X	X	X

[a] the day 180 visit will be a telephone visit

[b] if performed as part of standard of care at any time during hospitalization

[c] if performed as part of standard of care at admission

[d] in the natriuresis guided group

3.2 Screening

At screening the following assessments will be executed (all part of standard of care):

- Deferred informed consent
- Demographic data, including cardiovascular risk factors
- Medical history by chart review
- Vital signs
- Physical examination, including weight, height (only at screening), oedema, rales, jugular venous pressure (JVP), orthopnoea assessment, New York Heart Association (NYHA) class
- 12-lead electrocardiogram (ECG)
- NYHA class assessment
- Laboratory assessments, including haematology, renal function, NT-proBNP
- Concomitant medication
- In- and exclusion criteria

3.3 Assessment 1 (Baseline)

After having confirmed eligibility by confirming all inclusion criteria and by ruling out either one of the exclusion criteria, the following assessments will be executed:

- Randomization
- Vital signs
- Physical examination, including weight, oedema, rales, JVP, orthopnoea assessment, NYHA class
- Concomitant medication
- Initial loop diuretic dose in both groups will be determined based on several patients characteristics described in more detail in section 3.14.1 and figure 3
- Collection of EDTA blood sample for biomarker analysis and collection of spot urine sample for biomarker analysis
- Start fluid balance by noting ingested and excreted fluid volumes

3.4 Assessment 2 (2 hours)

At two hours, in patients randomized to the natriuresis guided group, the following assessments will be executed:

- Vital signs
- Evaluate Diuretic Response:
    - Spot urine sodium

        - If applicable adjust loop diuretic therapy based on this result (please see also section 3.14.2, and figure 4)
- Loop diuretic dose
- Collection of spot urine sample for biomarker analysis (in both groups)
- Diuresis assessment

3.5 Assessment 3.0 (6 hours)

At six hours the following assessments will be executed:

- Vital signs
- Physical examination, including oedema, rales, JVP, orthopnoea assessment, NYHA class
- Spot urinary sodium (result will be blinded in the SOC group)
- 6 hours urine collection
- In the Natriuresis Guided Group:
    - Evaluate Diuretic Response based on spot urinary sodium and diuresis and adjust loop diuretic therapy based on these results (please see also section 3.14.2, and figure 4)
- Loop diuretic dose
- Laboratory assessments, including haematology, and kidney function
- Collection of EDTA blood sample for biomarker analysis and collection of spot urine sample for biomarker analysis
- Diuresis assessment

3.6 Assessment 3.1 (12 hours)

In the natriuresis guided group the following assessments will be executed:

- Vital signs
- Evaluate Diuretic Response based on spot urinary sodium and diuresis and adjust loop diuretic therapy based on these results (please see also section 3.14.2, and figure 4)
- Loop diuretic dose

3.7 Assessment 3.2 (18 hours)

In the natriuresis guided group the following assessments will be executed:

- Vital signs

- Evaluate Diuretic Response based on spot urinary sodium and diuresis and adjust loop diuretic therapy based on these results (please see also section 3.14.2, and figure 4)
- Loop diuretic dose

3.8 Assessment 4.0 (24 hours)

At twenty-four hours the following assessments will be executed:

- Written informed consent (if not yet provided)
- Concomitant medication
- Vital signs
- Physical examination, including weight, oedema, rales, JVP, orthopnoea assessment, NYHA class
- Spot urinary sodium (result will be blinded in the SOC group)
- 24 hours urine collection
- In the Natriuresis Guided Group:
    - Evaluate Diuretic Response based on spot urinary sodium and diuresis and adjust loop diuretic therapy based on these results (please see also section 3.14.2, and figure 5)
- Loop diuretic dose
- Laboratory assessments, including haematology, and kidney function
- End of first timed twenty-four hour urine collection
- Collection of EDTA blood sample for biomarker analysis and collection of spot urine sample for biomarker analysis
- Diuresis and first 24 hour fluid intake assessment
- (Serious) adverse events

3.9 Assessment 4.1 (36 hours)

In the natriuresis guided group the following assessments will be executed:

- Vital signs
- Evaluate Diuretic Response based on spot urinary sodium and diuresis and adjust loop diuretic therapy based on these results (please see also section 3.14.2, and figure 5)
- Loop diuretic dose

3.10 Assessment 5 (48 hours)

At forty-eight hours the following assessments will be executed:

- Written informed consent (if not yet provided)
- Concomitant medication
- Vital signs
- Physical examination, including weight, oedema, rales, JVP, orthopnoea assessment, NYHA class
- Loop diuretic dose
- Laboratory assessments, including haematology, NT-proBNP, and kidney function
- End of second timed 24 hour urine collection
- Collection of EDTA blood sample for biomarker analysis and collection of spot urine sample for biomarker analysis
- Diuresis and second 24 hour fluid intake assessment
- (Serious) adverse events

3.11 Assessment 6 (72 hours)

At seventy-two hours the following assessments will be executed:

- Written informed consent (if not yet provided) – last opportunity
- Concomitant medication
- Vital signs
- Physical examination, including weight, oedema, rales, JVP, orthopnoea assessment, NYHA class
- Loop diuretic dose
- Laboratory assessments, including haematology, NT-proBNP, and kidney function
- End of third and final timed 24 hour urine collection
- Collection of EDTA blood sample for biomarker analysis and collection of spot urine sample for biomarker analysis
- Diuresis and third 24 hour fluid intake assessment
- (Serious) adverse events

3.12 Assessment 7 (discharge)

At discharge the following assessments will be executed:

- Concomitant medication
- Vital signs
- Physical examination, including weight, oedema, rales, JVP, orthopnoea assessment, NYHA class
- Loop diuretic dose
- Laboratory assessments, including haematology, NT-proBNP, and kidney function

- Collection of EDTA blood sample for biomarker analysis and collection of spot urine sample for biomarker analysis
- Diuresis and first 24 hour fluid intake assessment
- (Serious) adverse events

3.13 Assessment 8 (180 days +/- 10 days)

On day 180 the following assessments will be executed by phone call.

- Concomitant medication
- Weight (patient reported) and NYHA class
- Loop diuretic dose
- Heart failure admissions and/or mortality
- (Serious) adverse events

3.14 Treatment algorithm

Patients presenting with acute heart failure will be randomized to natriuresis guided therapy or standard of care (figure 2). When patients are randomized to the natriuresis guided treatment arm, decongestive treatment will be adjusted based on the spot urine sodium values assessed at set time points. The first natiuresis assessment takes place two hours after randomization. Patients will be required to empty the bladder at randomization (if deemed feasible), and a baseline urine sample will be obtained. In patients with a catheter the first sample (after placement of the urinary catheter) will be discarded. At two hours, a spot urinary sample will be obtained in patients with a urinary catheter after voiding the collected urine output in a container and obtaining the first produced urine after this. Patients without a catheter will be encouraged to urinate at this time point. This method will be repeated at the consecutive time points.

Figure 2: Overview of the treatment algorithm

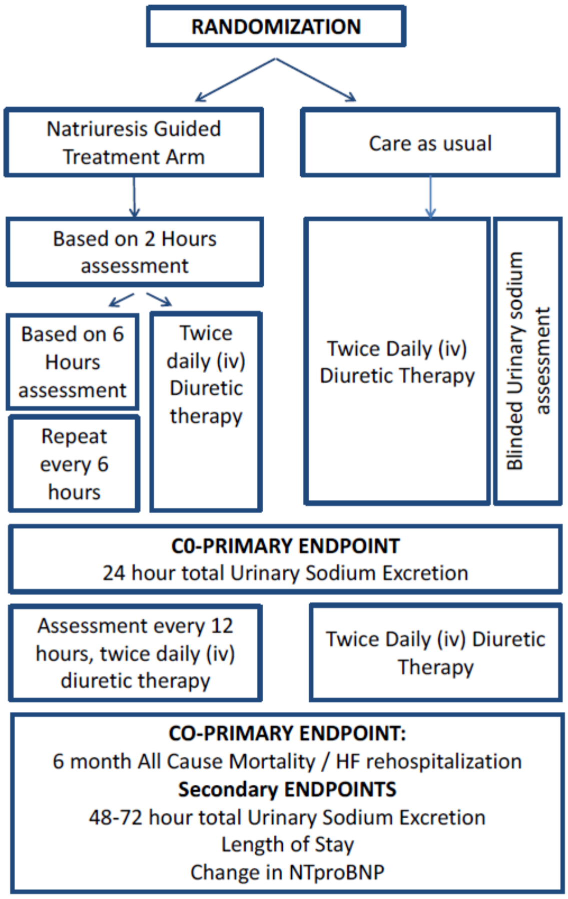

Abbreviations: HF: heart failure, iv: intravenously, LD: loop diuretic, NTproBNP: N-terminal pro blood natriuretic peptide

3.14.1 Loop diuretic dose at baseline

Baseline loop diuretic dose will be determined based on the renal function of the patient and his/her outpatient loop diuretic dose (figure 3). If available, bumetanide will be used, as this has better bioavailability than furosemide. Conversion from furosemide to bumetanide doses will be done by dividing the furosemide dose by 40. If bumetanide is not in stock at the UMCG, furosemide will be used.

If patients use loop diuretics in the outpatients setting and have a preserved renal function (estimated glomerular filtration rate (eGFR) ≥ 60 ml/min/1.73m^2), starting intravenous loop diuretic dose will be the total daily outpatient dose as a bolus and consequently continued in twice daily dosing.

If patients use loop diuretics in the outpatient setting and have an impaired renal function (eGFR < 60 ml/min/1.73m^2), starting intravenous loop diuretic dose will be twice the total daily outpatient dose as a bolus and this consequently continued in twice daily dosing.

If patients do not use loop diuretics in the outpatient setting (naïve), the loop diuretic dose will only be determined based on the renal function. In case of a preserved renal function a starting dose of 1 mg bumetanide will be administered which will be continued in twice daily dosing. When renal function is impaired a starting loop diuretic dose of 2 mg bumetanide will be used, which will consequently be continued in twice daily dosing.
In all groups, the maximum bolus dose will be 5 mg of bumetanide (200 mg of furosemide) as an additional effect above this initial bolus dose is not expected.

The determination of the loop diuretic dose at baseline is further illustrated in the following figure:

Figure 3: Determination of the starting loop diuretic dose in all patients

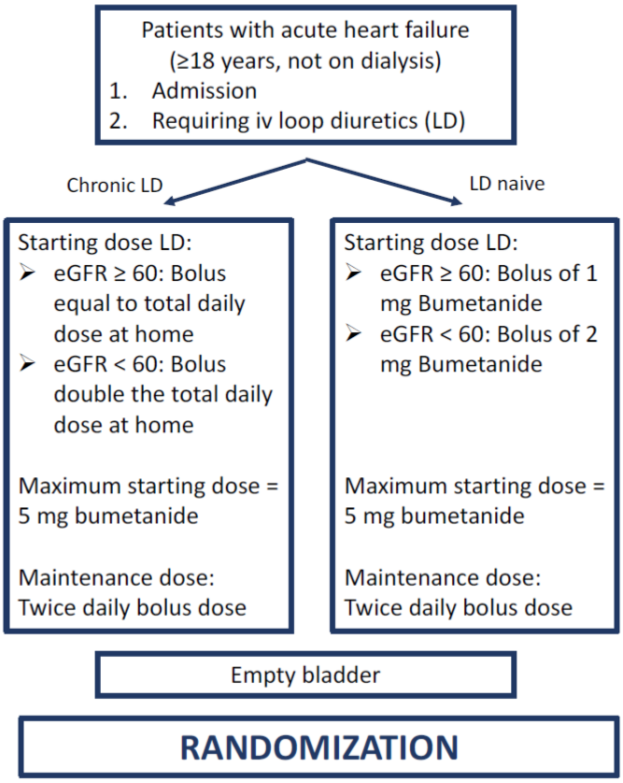

Abbreviations: eGFR: estimated glomerular filtration rate, iv: intravenously, LD: loop diuretic

Patients will be randomized using Epic in a 1:1 ratio to natriuresis guided therapy or standard of care.

3.14.2 Standard of care group

In the standard of care group decongestive treatment will be performed as it is currently done in clinical practice at the UMCG. After randomization patients will receive twice daily loop

diuretic doses intravenously, as previously described (figure 2 and 3). The maximum starting dose will be 5 mg of bumetanide (200 mg of furosemide) twice daily.

3.14.3 Natriuresis determined treatment algorithm

Based on the urinary sodium value obtained from 2 hours onwards in the natriuresis guided group, decongestive therapy will be adjusted using the following treatment algorithm.

Figure 4: Treatment algorithm for the natriuresis guided group during the first 24 hours after randomization

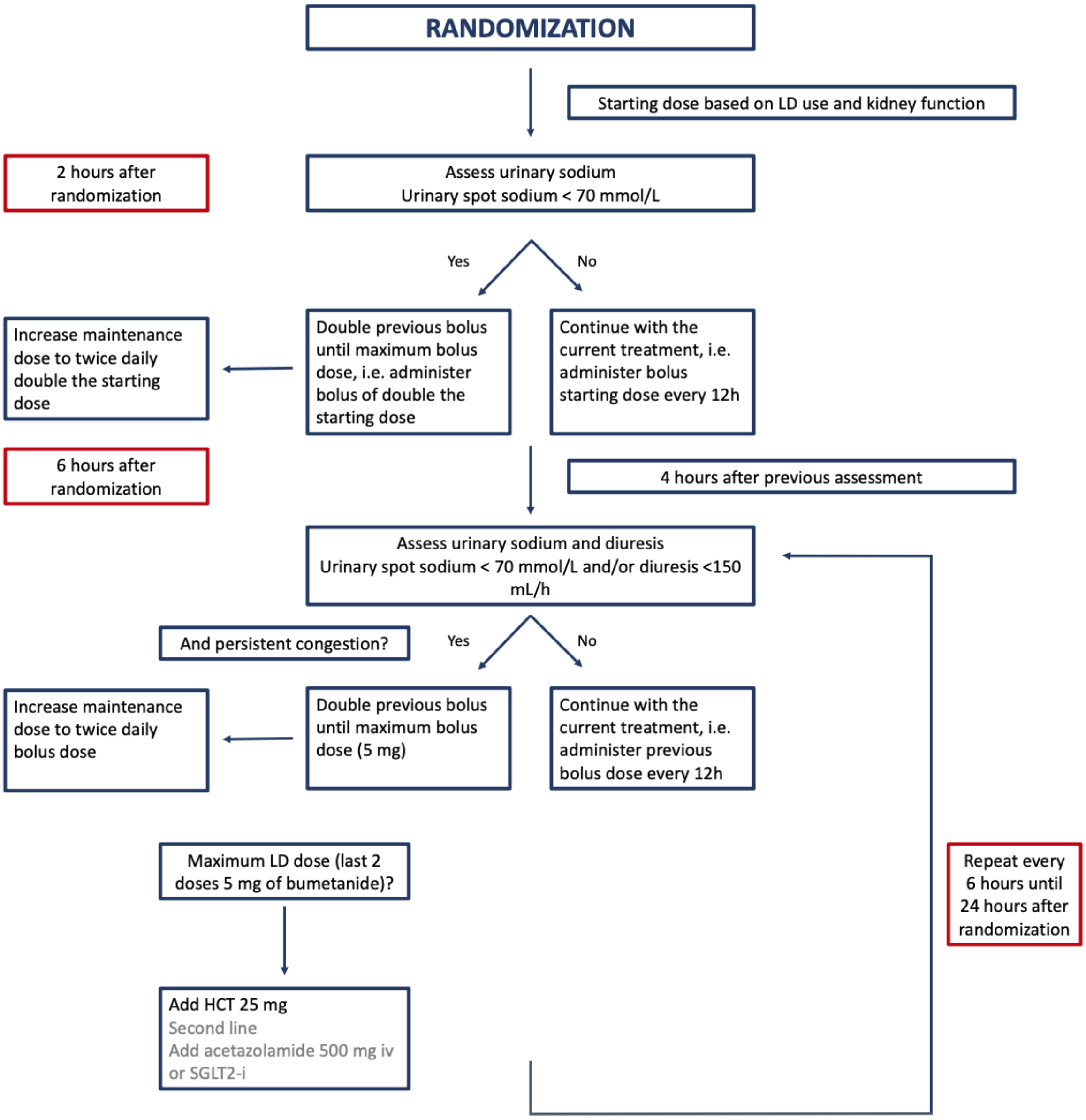

Abbreviations: HCT: hydrochlorothiazide, LD: loop diuretic, SGLT2-I (sodium glucose transporter 2 inhibitor)

In patients randomized to natriuresis guided therapy, the first urinary sodium will be determined at 2 hours. All urinary sodium assessments will be spot urinary samples. These will be obtained from the urinary catheter (if applicable) at set time points. If spot urinary sodium at 2 hours is < 70 mmol/L, patients will receive an additional bolus of loop diuretic, which will be double the previous bolus with a maximum bolus dose of 5 mg of bumetanide or equivalent (figure 4). If the bolus dose is doubled, the maintenance dose of loop diuretics will be adjusted to twice daily the doubled bolus dose (figure 4). If a patient already received 5 mg of bumetanide as a first dose, this dose will be repeated when there is insufficient natriuresis at this time point.

The next urinary sodium assessment is at 6 hours. At this time point physicians are required to assess both urinary sodium, and urine output. If urinary sodium is < 70 mmol/L, and/or diuresis is < 150 mL/hour, patients will be eligible (if still deemed congested) for an additional dose of loop diuretic of double the previous dose (figure 4) with a maximum bolus dose of 5 mg of bumetanide or equivalent. The maintenance dose will consequently be adjusted to twice daily the doubled bolus dose. Again, if a patient already received 5 mg of bumetanide as a first dose, this dose will be repeated when there is insufficient natriuresis or diuresis at this time point. Furthermore, if a patient has had two doses of 5 mg of bumetanide and has insufficient natriuresis or diuresis at two consecutive time points, the initiation of combination diuretic therapy with the addition of hydrochlorothiazide 25 mg once daily is indicated. If patients at admission already use a thiazide diuretic, yet no loop diuretic, the thiazide diuretic will be stopped upon admission. These patients are still eligible for combination diuretic therapy with hydrochlorothiazide and a loop diuretic. If patients intentionally use combination diuretic therapy at admission this will be continued. These patients will qualify for second step combination therapy with either acetazolamide or a sodium-glucose-cotransporter 2 inhibitor (SGLT2i).

The above protocol will be repeated at 12, 18, and 24 hours. When a patients has insufficient natriuresis or diuresis at two consecutive time points after the addition of hydrochlorothiazide, acetazolamide 500 mg iv once daily will be added. If this is contra-indicated or acetazolamide has been added as a first step for combination diuretic therapy, the addition of a sodium-glucose transporter 2 inhibitor should be considered.

The treatment algorithm will be continued until 48 hours, however after 24 hours natriuresis and diuresis assessment will only take place every 12 hours (figure 5). At every time point it is essential for physicians to only administer additional doses of diuretics if a patients is still congested. Combination diuretic therapy will be stopped when the patient is euvolemic or when this is indicated based on significant electrolyte disturbances or renal function decline in the absence of decongestion.

Figure 5: Treatment algorithm during the second 24 hours after randomization

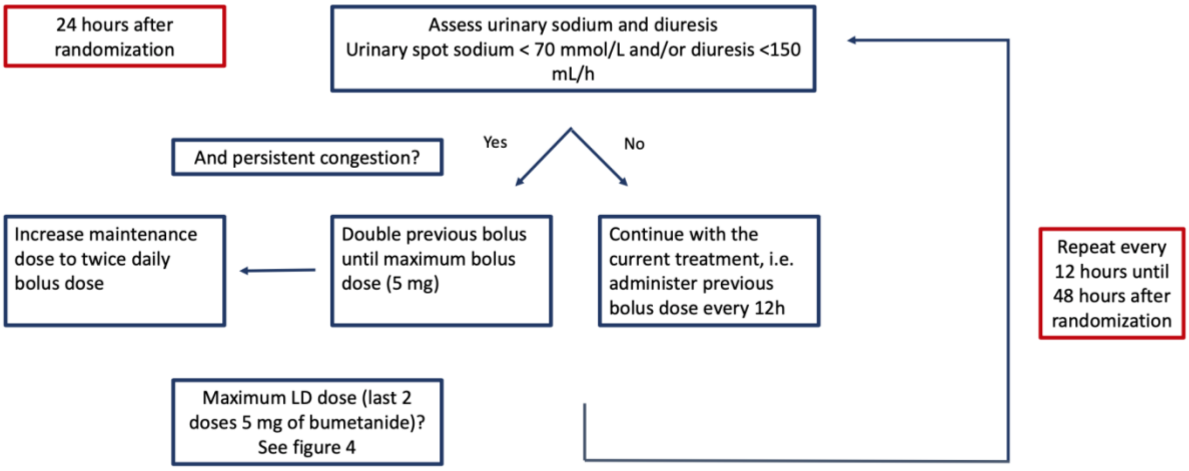

Abbreviations: LD: loop diuretic

3.15 Renal ultrasound substudy

3.15.1 Introduction and rationale ultrasound substudy

It is well known that assessment of congestion status remains difficult, as clinical symptoms and signs of HF are in part difficult to assess, have high inter-observer variability, and are mostly non-specific. Non-invasive technologies such as ultrasound imaging to assess congestion and fluid status could aid in the (early) clinical identification of these HF signs and symptoms, and detect change in congested state as response to diuretic treatment.

Ultrasound measurements are easily accessible, interpretable, non-invasive, inexpensive, safe, and can be performed at the bedside rapidly providing additional information and therefore might improve diagnosis and treatment in HF patients. Moreover, it may help detect patients in need of treatment (de)intensification to prevent (re-)admission for HF.

Therefore, patients enrolled during daytime hours on Monday till Wednesday will be eligible for participation in a substudy aimed at better assessment of congestion using ultrasound.

3.15.2 Objectives ultrasound substudy

Primary objective:

To assess congestion status through bedside renal, cardiac, and lung ultrasound assessments in patients with acute heart failure at baseline, day 1, day 2, day 3 and discharge, and change over time.

Secondary Objective(s):

To assess the effect of diuretic therapy, the effect of natriuresis guided therapy specifically, and the relationship between biomarkers of congestion and (change in) congestion status measured by serial ultrasound assessments (cardiac, renal, lung).

3.15.3 Study design ultrasound substudy

This will be a prospective, observational, exploratory study, in which we will evaluate renal, lung, and cardiac ultrasound measurements at baseline (day 1), day 2, day 3 and at day of discharge. Patients will be enrolled from the primary study, with corresponding inclusion and exclusion criteria. All patients will provide additional informed consent for enrollment in the substudy. Patients will be included on week days during day time hours on Monday, Tuesday or Wednesday. We aim to enroll a total of 50 patients and will strive for equal distribution between the natriuresis and the standard of care group of the primary study. Treating physicians will be blinded to the results of the ultrasound assessments. Ultrasounds will be performed by trained clinicians.

Study population

In a proof-of-concept study we performed in patients with acute heart failure, we observed that 54% of patients had a normalization of renal venous flow after 3 days of decongestive therapy. We expect this to increase to approximately 80% of patients in the natriuresis guided group. With an alpha of 0.05 and a power of 80%, we require 48 patients for this study, which we will increase to 50 patients to prevent being underpowered for instance due to insufficient image quality.

Study visits

Table 3.15. Assessment schedule ultrasound substudy

Assessments	Baseline (day 1)	Day 2	Day 3	Discharge
Renal ultrasound<br>- Renal venous flow pattern<br>- Venous impedance index<br>- Venous discontinuity index<br>- Intrarenal arterial resistance index	X	X	X	X
Transthoracic echocardiogram<br>- Inferior vena cave diameter<br>- Right ventricular peak pressure	X	X	X	X

- Pulmonary vein flow				
Lung ultrasound<br>- B-lines	X	X	X	X

Statistical analysis

Baseline data will be presented using mean +- standard deviation for normally distributed variables, mean (interquartile ranges) for skewed variables, and as frequencies (percentages) for categorical variables. Participants will be divided into groups according to occurrence of change in renal, cardiac or lung ultrasound measurements. Baseline characteristics will be analyzed using t-test, linear regression models or Cuzick's nonparametric test for continuous variables, and chi-square test for categorical variables. Predictors at baseline for change in ultrasound measurements will be determined using regression models.

3.16 Pharmacodynamic substudy

In a subset of patients we will perform a pharmacodynamic study aimed at gaining a better understanding of the effect of a bolus of intravenous diuretics on sodium excretion trajectory in contemporary acute heart failure patients. At the moment the only data regarding this stems from the eighties and was obtained in patients with chronic heart failure. This pharmacodynamic substudy can be performed after an intravenous dose of loop diuretics during daytime hours before day 3. Only patients with a urinary catheter will be eligible for this substudy. In addition to the urine samples already obtained per protocol at baseline, after 2 hours and after 6 hours, additional urinary spot samples will be obtained every 30 minutes during the first 6 hours (12 urine samples). One additional plasma samples will be obtained. All patients will provide additional informed consent for enrollment in the pharmacodynamic substudy.

Study population

With an estimate of Urinary sodium content of 90 +/- 27 mmol/L after 2 hours, with 95% CI, and making sure that what we measure is within 12 mmol/L of the true population (margin of error), the formula will be as follows $N = (1.96(27)/12)^2$. This results in approximately 20 patients to ensure that the 95% CI of 2 hour Urinary sodium concentration is within 12 mmol/L of the true mean. Therefore, we aim to enroll 20 patients in this substudy.

4. STUDY POPULATION

4.1 Population (base)

The study population consist of male and female patients (≥18 years old) admitted to the hospital for acute heart failure and requiring intravenous diuretic therapy at the University Medical Centre Groningen.

4.2 Inclusion criteria

In order to be eligible to participate in this study, a subject must meet all of the following criteria:

1. Male or female ≥ 18 years of age
2. Primary diagnosis of acute /decompensated heart failure as assessed by treating physician
    a. Acute Heart failure can be de novo or exacerbation of known heart failure and diagnosis is based on criteria in the ESC HF guidelines
3. Requirement of intravenous diuretic use

4.3 Exclusion criteria

A potential subject who meets any of the following criteria will be excluded from participation in this study:

1. Dyspnoea primarily due to non-cardiac causes
2. Patients with severe renal impairment receiving dialysis or requiring ultrafiltration
3. Inability to follow instructions
4. Previous participation in this study
5. Any other medical conditions that may put the patient at risk or influence study results in the investigator's opinion, or that the investigator deems unsuitable for the study

4.4 Sample size calculation

Based on our previous study in acute heart failure patients, the mean 24 hours sodium excretion was 398 ± 246 mmol.(8; METC 2019/437) In this population, not 2 hours but 6 hours measurements were available, and in these patients, 36% of patients had an insufficient response, defined as urinary sodium <90 mmol or urine output <900 mL at 6 hours. Assuming a 40% improvement in these 36% of patients, and a conservative 15% improvement in the remaining patients in their 24-hours urinary sodium excretion because of

closer monitoring, this will assume an overall 24% improvement in 24-hours urinary sodium excretion (0.36*1.40 + 0.64*1.15). Therefore, to obtain a power of at least 80% at a two-sided significance level of 0.025 (Bonferroni correction), we calculated a sample size of 125 patients in each group would be sufficient for the primary endpoint of 24-hour natriuresis. To prevent being underpowered due to drop-out or missing data, which is expected to be higher than average in this patient population and given the delicate nature of urinary collections, we will increase enrolment by 10% therefore requiring 140 patients per group, and enrolling a total of 280 patients.

Based on this sample size, we will have 81% power with a two-sided significance level of 0.025 to detect a HR of 0.49 for the other co-primary endpoint of all-cause mortality and heart failure rehospitalisation at 6 months (a reduction in events from 38% to 21%). However also accounting for 10% missing follow-up data, we will increase the total number of patients to 310 (155 patients per group).

5. TREATMENT OF SUBJECTS

The aim of this study is to establish the value of *natriuresis guided therapy* in acute heart failure, to *improve diuretic response, decongestion, and clinical outcomes*.

Patients will be randomized to either standard of care *or* natriuresis guided therapy. In the natriuresis guided group treatment will be adjusted based on urinary sodium values determined at set time points. The optimization of decongestive treatment will be done using a prespecified treatment algorithm (figures 4 and 5). In the standard of care group physicians are encouraged to treat patients according to current best practice consensus (figure 2).

5.1 Investigational product/treatment

The treatment algorithm used in this study is described in detail in section 3.14.2 and figures 4 and 5.

5.2 Use of co-intervention (if applicable)

Not applicable

5.3 Escape medication (if applicable)

Not applicable

6. METHODS

6.1 Study parameters/endpoints

6.1.1 Main study parameter/endpoint

To establish the effect of natriuresis guided therapy in patients with acute heart failure on 24-hours natriuresis and first occurrence of all-cause mortality or heart failure rehospitalisation at 6 months.

6.1.2 Secondary study parameters/endpoints (if applicable)

Secondary outcomes:

- 48- and 72-hours natriuresis
- Length of hospital stay
- Percentage change in NT-proBNP at 48 and 72 hours

6.1.3 Other study parameters (if applicable)

Safety endpoints:

- Doubling of serum creatinine at 24 or 48 hours
- Occurrence of worsening heart failure (for definition see section 7.2.1.)
- Adverse and serious adverse events

6.2 Randomization, blinding and treatment allocation

Subjects will be randomized 1:1 to natriuresis guided therapy or standard of care. Randomization will be executed using Epic. Treatment assignment will take place by Epic. Patients will maintain this randomization number throughout the study.
Randomization will be achieved within the EPIC electronic health record (EHR) system using an internal random number rule. Randomization starts the first time the patients chart is opened by a provider from the department of cardiology. The randomization group is maintained as fixed variable in the EPIC EHR. Then, when treatment with loop diuretic therapy (intravenous) is started, based on this randomization number, the EPIC EHR automatically chooses the correct treatment orders and diagnostic tests based on the randomization arm. After enrollment of 150 patients we will perform an interim analysis to check whether the distribution of patients to groups is indeed 1:1. If necessary the internal random number rule will be adjusted after this interim analysis.

6.3 Study procedures

Study patients will not be required to come to the hospital for additional visits. Blood will be drawn at 6 different time points during hospitalization. These are assessments that are already part of standard of care and therefore will not lead to additional blood draws. At these time points an additional sample of 5 mL will be obtained for additional assessments after completion of the trial.

A timed collection of urine will take place for the first 72 hours, from which urine samples will be obtained at a maximum of 7 different time points (and one additional urine sample at discharge) in the interventional arm. At these time points an additional sample of 5 mL will be obtained for additional assessments after completion of the trial.

In these urine and plasma samples, we will determine biomarkers to further elucidate response to treatment by assessing congestion markers, and markers specific for certain segments of the kidney. This will not only provide more insight in possible mechanisms of diuretic response, but may also lead to identification of (new) targets for therapy.

Finally, a telephone call 180 days after baseline will be performed to assess signs and symptoms, weight as well as current medications. Vital status will first be verified by contacting the general practitioner.

See also table 3.1 for a schedule of assessments.

Study assessments as part of clinical care:

- Physical examination
  - Height, weight
  - Blood pressure, heart rate (vital signs)
  - Jugular venous pressure, heart sounds and murmurs, pulmonary rales, edema and ascites
- NYHA functional class heart failure
  - Class I: patients with no limitation of activities; they suffer no symptoms from ordinary activities
  - Class II: Patients with slight, mild limitation of activity; they are comfortable with rest or with mild exertion
  - Class III: Patients with marked limitation of activity; they are comfortable only at rest
  - Class IV: Patients who should be at complete rest, confined to bed or chair; any physical activity brings on discomfort and symptoms occur at rest
- HF signs and symptoms
  - Anamnesis: dyspnea d’effort, orthopnea, paroxysmal nocturnal dyspnea, edema, exercise tolerance

    - Physical examination (see above)
- Echocardiogram
    - Only if performed as standard of care during hospitalization
- X-ray
    - Only if performed as standard of care during hospitalization
- ECG
    - Only if performed as standard of care during hospitalization
- Standard laboratory analysis (standard of care)
    - At admission: hematology, renal function, electrolytes, NT-proBNP
    - After 6 hours: renal function, electrolytes, NT-proBNP
    - Daily: hematology, renal function
- Fluid intake assessment

6.4 Withdrawal of individual subjects

Subjects can leave the study at any time for any reason if they wish to do so without any consequences. The investigator can decide to withdraw a subject from the study for urgent medical reasons.

6.4.1 Specific criteria for withdrawal (if applicable)

Not applicable

6.5 Replacement of individual subjects after withdrawal

Withdrawn subjects will not be replaced.

6.6 Follow-up of subjects withdrawn from treatment

Subjects withdrawn from treatment will be asked whether the investigator will be allowed to contact the patient for the 180-days visit in order to collect information regarding vital status and eventual (serious) adverse events. Investigators will be urged to have subjects consent to them gathering follow-up information of each withdrawn subject.

6.7 Premature termination of the study

The study may be prematurely terminated if the following criterion applies:

- Apparent inability to include sufficient subjects

7. SAFETY REPORTING

7.1 Temporary halt for reasons of subject safety

In accordance to section 10, subsection 4, of the WMO, the sponsor will suspend the study if there is sufficient ground that continuation of the study will jeopardise subject health or safety. The sponsor will notify the accredited METC without undue delay of a temporary halt including the reason for such an action. The study will be suspended pending a further positive decision by the accredited METC. The investigator will take care that all subjects are kept informed.

7.2 AEs, SAEs and SUSARs

7.2.1 Adverse events (AEs)

Adverse events are defined as any undesirable experience occurring to a subject during the study, whether or not considered related to the intensified treatment. All adverse events reported spontaneously by the subject or observed by the investigator or his staff will be recorded. All (serious) adverse events will be recorded from baseline to the final follow-up visit (phone call) at 180 days.

Pre-specified adverse events are defined as follows:

- Worsening heart failure defined as:
  - Addition or starting of inotropes or vasopressors
  - Mechanical ventilation
  - Palliative care due to progressive heart failure
  - Any intervention/treatment for heart failure that leads to prolonged hospitalization
- True worsening renal function
  - Doubling of creatinine from baseline to 48 or 72 hours without evidence of decongestion, or urine production < 10 cc / hour despite adequate dosing of loop diuretics

7.2.2 Serious adverse events (SAEs)

A serious adverse event is any untoward medical occurrence or effect that

- results in death;
- is life threatening (at the time of the event);
- requires hospitalization or prolongation of existing inpatients' hospitalization;
- results in persistent or significant disability or incapacity;

- is a congenital anomaly or birth defect; or
- any other important medical event that did not result in any of the outcomes listed above due to medical or surgical intervention but could have been based upon appropriate judgment by the investigator.

An elective hospital admission will not be considered as a serious adverse event.

The investigator will report all SAEs to the sponsor without undue delay after obtaining knowledge of the events..

The sponsor will report the SAEs through the web portal *ToetsingOnline* to the accredited METC that approved the protocol, within 7 days of first knowledge for SAEs that result in death or are life threatening followed by a period of maximum of 8 days to complete the initial preliminary report. All other SAEs will be reported within a period of maximum 15 days after the sponsor has first knowledge of the serious adverse events.

7.2.3 Suspected unexpected serious adverse reactions (SUSARs)

Not applicable

7.3 Annual safety report

Not applicable.

7.4 Follow-up of adverse events

All AEs will be followed until they have abated, or until a stable situation has been reached. Depending on the event, follow up may require additional tests or medical procedures as indicated, and/or referral to the general physician or a medical specialist.
SAEs need to be reported till end of study.

7.5 Endpoint adjudication committee

This committee of experts will adjudicate all rehospitalizations to judge whether a hospitalization is due to heart failure. The committee will be blinded to the treatment allocation, and will independently review each case in order of appearance. Their independent opinions are reconciled at the endpoint adjudication committee meeting. If an endpoint is not unanimous, the case is reviewed further and discussed with the aim of developing a consensus. If the committee is unable to reach a consensus, the endpoint will be established by the committee chair.

A hospitalization for heart failure will be defined as follows:
Hospitalization for more than one day (change in calendar day) with an exacerbation of heart failure requiring treatment meeting the following criteria:

- Signs and symptoms of heart failure
    - One or more of the following symptoms consistent with heart failure:
        - Dyspnea
        - Orthopnea
        - Paroxysmal nocturnal dyspnea
        - Increasing fatigue/decreasing exercise tolerance
        - Edema/anasarca
        - Other symptoms of worsened end-organ perfusion such as dizziness, mental confusion
    - AND two or more of the following sign consistent with heart failure:
        - Weight gain
        - Pulmonary edema or rales
        - Elevated jugular venous pressure
        - Radiologic signs of heart failure
        - Peripheral edema
        - Abdominal distension or ascites
        - S3 gallop rhythm
        - Positive hepatojugular reflux
        - Elevated NT-proBNP
        - Congestive hepatomegaly
        - Invasive/non-invasive tests showing elevated cardiac filling pressures or low cardiac output
- AND treatment
    - The patient receives initiation or intensification of treatment specifically for heart failure,

STATISTICAL ANALYSIS

7.6 Primary study parameter(s)

The primary endpoint will be assessed in the intention to treat (ITT) population. The co-primary endpoint will be total 24-hour natriuresis and first occurrence of all-cause mortality or heart failure rehospitalisation at 6 months. Following the Bonferroni correction used for the sample size calculation, the study will be deemed positive if one or both of the components of the co-primary endpoint are positive (*P-value*<0.025).

24-hour natriuresis will be calculated and presented as mean +/- SD if normally distributed, or median (25th and 75th percentile) in the case of non-normal distribution. The between group difference will be tested using t-test if normally distributed, or Wilcoxon rank sum test if non-normally distributed. The effect of natriuresis guided treatment on long-term outcomes will be assessed using Cox regression for the between treatment difference.

For the presentation of baseline characteristics in both treatment arms, continuous variables will be presented as mean +/- SD, non-normally distributed variables as median (25th – 75th percentile), and categorical values as count (percentages).

7.7 Secondary study parameter(s)

Secondary response variables include 48- and 72-hour natriuresis, length of hospital stay and percentage change in NT-proBNP at 48- and 72-hours. Changes in biomarkers, natriuresis at subsequent time points, and length of hospital stay will be assessed using t-test for normal distributed variables, and Wilcoxon rank sum test if non-normally distributed.

7.8 Other study parameters

Adverse event rates, including doubling of serum creatinine at 24 or 48 hours, serious adverse events, as well as worsening heart failure will be analyzed using Fisher's exact tests, and presented as counts (percentages). To study differences in repeated assessments between treatment groups, such as natriuresis during hospitalization, repeated measures linear mixed effect modeling will be used.

7.9 Interim analysis (if applicable)

Not applicable

8. ETHICAL CONSIDERATIONS

8.1 Regulation statement

This clinical study was designed and shall be implemented and reported in accordance with the guidelines for Good Clinical Practice, with applicable local regulations, and with the ethical principles held down in the Declaration of Helsinki (date 19-10-2013). This study will be conducted in accordance with the medical Research involving Human Subjects Act (WMO). The guidelines mentioned in WMO will be the principle guidelines.
An institutional review board that complies with the requirements of applicable Law shall review and approve this protocol, including the informed consent form, in accordance with Applicable Law.

8.2 Recruitment and consent

Recruitment of patients will take place at the Emergency Department, Coronary Care Unit or Ward of the department of Cardiology of the University Medical Center Groningen. Once a potentially eligible patient is diagnosed with acute heart failure, the Principal Investigator or a sub-investigator (physician) will verify eligibility based on the in- and exclusion criteria. Once eligibility has been established, the patient will be enrolled. Because of the acute situation and the nature of the study treatment, there is limited time (at that moment) for the patient to consider participation, therefore deferred consent will be used. At this time treatment will start, as well as collection of urine. The patient will receive the patient information letter about the study during the first four days of hospitalization and will have a maximum of 24 hours to consider his/her participation. During this time written informed consent will be obtained by the researcher.

.

10.3 Objection by minors

8.3 Objection by minors or incapacitated subjects (if applicable)

Not applicable.

8.4 Benefits and risks assessment, group relatedness

Loop diuretics are the cornerstone of treatment in patients with acute heart failure. Loop diuretics are relatively safe and well tolerated by patients in acute heart failure. There is a wealth of data describing its use in this patient group, as well as in patients with a decreased kidney function. Combination of loop diuretics with other diuretic agents, such as acetazolamide has been shown to be able to improved decongestion. Risks of combination diuretic therapy are electrolyte disorders and decrease in kidney function. We will therefore

only initiate combination diuretic therapy in patients with insufficient decongestion already on maximal loop diuretic doses. We will additionally closely monitor renal function and electrolytes throughout the study. Furthermore, assessment of natriuresis is at low risk and minimal hassle to the patient as it does not involve additional invasive procedures. Based on the previous, the relative risk is estimated to be relatively low, while potential benefit may be expected.

8.5 Compensation for injury

The sponsor/investigator has a liability insurance which is in accordance with article 7 of the WMO.

The sponsor (also) has an insurance which is in accordance with the legal requirements in the Netherlands (Article 7 WMO). This insurance provides cover for damage to research subjects through injury or death caused by the study.
The insurance applies to the damage that becomes apparent during the study or within 4 years after the end of the study.

8.6 Incentives

Patients will not receive any incentives, compensations or treatment through participation in the study.

9. ADMINISTRATIVE ASPECTS, MONITORING AND PUBLICATION

9.1 Handling and storage of data and documents

A subject identification code list will be made to link the data to the subject in order to be able to trace data to an individual subject. This code will not be based on the patients initials and birth date. The key to the code will be safeguarded by the investigator since the data will be kept for a period of 15 years. The handling of personal data will comply with privacy laws, legislation, codes and/or guidelines that apply in the applicable jurisdictions the study is conducted.

9.2 Monitoring and Quality Assurance

Independent monitors will monitor the study according to a pre-specified monitoring plan. The monitors are trained in GCP and will be trained on study specific tasks and processes. As part of the UMCG Quality Management Strategy monitor oversight will be implemented through regular documentation reviews and co-monitoring activities.

9.3 Amendments

All substantial amendments will be notified to the METC and to the competent authority. Non-substantial amendments will not be notified to the accredited METC and the competent authority, but will be recorded and filed by the sponsor.

9.4 Annual progress report

The sponsor/investigator will submit a summary of the progress of the trial to the accredited METC once a year. Information will be provided on the date of inclusion of the first subject, numbers of subjects included and numbers of subjects that have completed the trial, serious adverse events/ serious adverse reactions, other problems, and amendments.

9.5 Temporary halt and (prematurely) end of study report

The sponsor will notify the accredited METC and the competent authority of the end of the study within a period of 90 days. The end of the study is defined as the last patient's last visit.

In case the study is ended prematurely, the sponsor will notify the accredited METC and the competent authority within 15 days, including the reasons for the premature termination.

Within one year after the end of the study, the investigator/sponsor will submit a final study report with the results of the study, including any publications/abstracts of the study, to the

accredited METC and the Competent Authority.

9.6 Public disclosure and publication policy

The trial will be registered in a public trial registry. The results will be disclosed unreservedly.

10. STRUCTURED RISK ANALYSIS

10.1 Potential issues of concern

Not applicable as the drugs in this study are used for their registered indication.

10.2 Synthesis

Loop diuretics are the cornerstone of treatment of acute heart failure by attempting to establish a negative sodium and consequently fluid balance. Loop diuretics inhibit the sodium-chloride-potassium co-transporter in the thick ascending loop of Henle and as such lead to decreased sodium and chloride reabsorption from the urine. There is ample data on loop diuretic use in acute heart failure patients as these are administered to >90% of patients hospitalization for acute heart failure. Loop diuretics currently have a class 1C indication in the heart failure guidelines to improve symptoms. All of the drugs used in the study have been used extensively in clinical trials of patients with (acute) heart failure and have been granted approval for their use in heart failure patients.

In this study adverse events of special interest are collected. These include worsening heart failure and true worsening renal function. These side effects are rare but will be monitored closely by regular monitoring of clinical status and renal function. The potential benefits of natriuresis guided therapy to improve the treatment of acute heart failure clearly outweigh the risk of developing these adverse events.

11. REFERENCES

1. www.volksgezondheidzorg.info/onderwerp/hartfalen.
www.volksgezondheidzorg.info/onderwerp/hartfalen

2. Ter Maaten JM, Valente MA, Damman K, Hillege HL, Navis G, Voors AA. Diuretic response in acute heart failure-pathophysiology, evaluation, and therapy. *Nat Rev Cardiol* 2015;**12**:184-192.

3. Testani JM, Brisco MA, Turner JM, Spatz ES, Bellumkonda L, Parikh CR, Tang WH. Loop Diuretic Efficiency: A Metric of Diuretic Responsiveness with Prognostic Importance in Acute Decompensated Heart Failure. *Circ Heart Fail* 2013;

4. Valente MA, Voors AA, Damman K, Van Veldhuisen DJ, Massie BM, O'Connor CM, Metra M, Ponikowski P, Teerlink JR, Cotter G, Davison B, Cleland JG, Givertz MM, Bloomfield DM, Fiuzat M, Dittrich HC, Hillege HL. Diuretic response in acute heart failure: clinical characteristics and prognostic significance. *Eur Heart J* 2014;

5. Voors AA, Davison BA, Teerlink JR, Felker GM, Cotter G, Filippatos G, Greenberg BH, Pang PS, Levin B, Hua TA, Severin T, Ponikowski P, Metra M, for the RELAX-AHF Investigators. Diuretic response in patients with acute decompensated heart failure: characteristics and clinical outcome-an analysis from RELAX-AHF. *Eur J Heart Fail* 2014;

6. ter Maaten JM, Dunning AM, Valente MAE, Damman K, Ezekowitz JA, Califf RM, Starling RC, van der Meer P, O'Connor CM, Schulte PJ, Testani JM, Hernandez AF, Tang WHW, Voors AA. Diuretic response in acute heart failure - an analysis from ASCEND-HF. *American Heart Journal* 2015;

7. Ter Maaten JM, Valente MA, Damman K, Cleland JG, Givertz MM, Metra M, O'Connor CM, Teerlink JR, Ponikowski P, Bloomfield DM, Cotter G, Davison B, Subacius H, van Veldhuisen DJ, van der Meer P, Hillege HL, Gheorghiade M, Voors AA. Combining Diuretic Response and Hemoconcentration to Predict Rehospitalization After Admission for Acute Heart Failure. *Circ Heart Fail* 2016;**9**:10.1161/CIRCHEARTFAILURE.115.002845.

8. Damman K, Ter Maaten JM, Coster JE, Krikken JA, van Deursen VM, Krijnen HK, Hofman M, Nieuwland W, van Veldhuisen DJ, Voors AA, van der Meer P. Clinical importance of urinary sodium excretion in acute heart failure. *Eur J Heart Fail* 2020;

9. Testani JM, Hanberg JS, Cheng S, Rao V, Onyebeke C, Laur O, Kula A, Chen M, Wilson FP, Darlington A, Bellumkonda L, Jacoby D, Tang WH, Parikh CR. Rapid and Highly Accurate Prediction of Poor Loop Diuretic Natriuretic Response in Patients With Heart Failure. *Circ Heart Fail* 2016;**9**:e002370.

Statistical analysis plan PUSH-AHF

**Trial number**	NL75163.042.20<br>NCT04606927
**Title**	Pragmatic Urinary Sodium-based treatment algoritHm in Acute Heart Failure<br>PUSH-AHF
**Principle investigators**	J.M. ter Maaten and K. Damman
**Responsible trial statistician**	Dr D. Postmus
**Date of statistical analysis plan**	May 30th 2023
**Version**	1.0

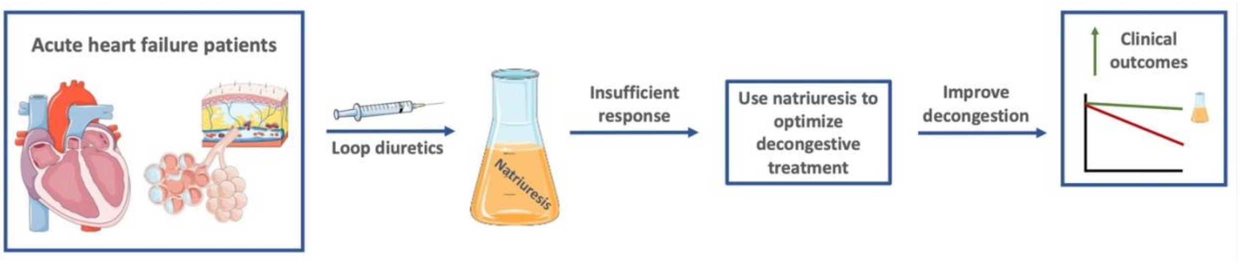

1. Document History

Version number	Version date	Author	Reason for change
Version 1.0	1.0	ter Maaten, Damman, Postmus	First version

2. Table of contents

1. Document history
2. Table of contents
3. List of abbreviations
4. Introduction
5. Study synopsis
   5.1 Study objectives and endpoints
      5.1.1 Rationale
      5.1.2 Primary endpoint
      5.1.3 Secondary endpoints in pre-specified hierarchical order
      5.1.4 Safety endpoints
      5.1.5 Exploratory endpoints
   5.2 Definitions
      5.2.1 End of study date
      5.2.2 Withdrawal of consent
      5.2.3 Cross-over from treatment allocation group
      5.2.4 Vital status
      5.2.5 Lost to follow-up
   5.3 Study design
      5.3.1 Randomization
      5.3.2 Blinding
      5.3.3 Number of patients
6. Analyses sets
7. Endpoint variables
   7.1 Dual-primary endpoint
   7.2 Secondary endpoints
8. Statistical analysis
   8.1 Closed testing procedure
   8.2 Analysis of the primary endpoint
   8.3 Secondary endpoint(s)
   8.4 Subgroup analysis
   8.5 Other study parameters
   8.6 Missing data
   8.7 Other sensitivity analysis
   8.8 Interim analysis (if applicable)
   8.9 Pre-specified sub-analyses
      8.9.1 Clinical outcome

8.9.2 Congestion status
8.9.3 Renal function
8.9.4 HF phenotype (HFrEF, HFmrEF, HFpEF)
8.9.5 Win ratio
8.9.6 Home diuretic use
8.9.7 Pharmacodynamic substudy
8.9.8 Renal ultrasound substudy
8.9.9 Combined analysis with ESCALATE

3 List of abbreviations

EAC	Endpoint Adjudication Committee
HF	Heart Failure
ICH	International Conference on Harmonisation
ITT	Intention To Treat
NT-proBNP	N Terminal pro Blood Natriuretic Peptide
PUSH-AHF	Pragmatic Urinary Sodium-based treatment algoritHm in Acute Heart Failure
SAP	Statistical Analysis Plan
SD	Standard Deviation

4 Introduction

As per the International Conference on Harmonisation (ICH) E9 guidance, the purpose of this document is to provide a more technical and detailed description of the analysis described in the protocol. The statistical analysis plan (SAP) assumes familiarity with the trial protocol, which may be consulted for more background information on the study. R version 4.3.0 will be used for all analyses.

5 Study synopsis

The Pragmatic Urinary Sodium-based treatment algoritHm in Acute Heart Failure (PUSH-AHF) trial is a pragmatic, single-center, randomized, controlled, open-label study, comparing natriuresis guided therapy with standard of care in patients with acute heart failure requiring treatment with intravenous loop diuretics.

5.1 Study objectives and endpoints

5.1.1 Rationale

Administration of loop diuretics to achieve decongestion is the current cornerstone of therapy for acute heart failure. Unfortunately, there is a lack of evidence on how to guide diuretic treatment. Recently, urinary sodium, as a measure of diuretic response, has been proposed as a target for therapy. The hypothesis of this study is that natriuresis guided therapy in patients with acute heart failure will improve diuretic response, and reduce long term clinical outcome.

5.1.2 Primary endpoint

The dual-primary endpoint is defined as: a) total 24-hour natriuresis and b) first occurrence of all-cause mortality or adjudicated heart failure (HF) rehospitalization at 6 months after randomization.

**Primary Endpoint:**	**Outcome Measure:**
To determine whether a natriuresis guided diuretic strategy can improve natriuresis and clinical outcomes (all cause death, HF rehospitalization)	1) Total Natriuresis at 24 hours after randomization<br>2) Time to the first occurrence of any of the components of this composite:<br>a. All cause Death<br>b. (Adjudicated) HF Rehospitalization

5.1.3 Secondary endpoints in prespecified hierarchical order

Key secondary endpoints are:

- Total 48-hour natriuresis (from 0-48 hours)
- Total diuresis at 24-hours (from 0-24 hours)
- Total diuresis at 48 hour (from 0-48 hours)
- Length of hospital stay from baseline to discharge
- HF rehospitalization: time to first event, total number of HF rehospitalizations (recurrent events)
- All-Cause Mortality

**Secondary Endpoints:**	**Outcome Measure:**
To compare the effect of a natriuresis guided diuretic strategy versus standard of care on total natriuresis after 48 hours	Total natriuresis (mmol) from randomization to 48 hours after randomization
To compare the effect of a natriuresis guided diuretic strategy versus standard of care on total diuresis after 24 hours	Total diuresis (mL) from randomization to 24 hours after randomization
To compare the effect of a natriuresis guided diuretic strategy versus standard of care on total diuresis after 48 hours	Total diuresis (mL) from randomization to 48 hours after randomization
To compare the effect of a natriuresis guided diuretic strategy versus standard of care on length of stay of the index hospitalization	Number of days in hospital for index HF admission
To compare the effect of a natriuresis guided diuretic strategy versus standard of care on total number of recurrent HF hospitalizations	Total number of (first and recurrent) HF hospitalizations
To compare the effect of a natriuresis guided diuretic strategy versus standard of care on all-cause mortality	Time to death from any cause
To compare the effect of a natriuresis guided diuretic strategy versus standard of care on percentage change in NT-proBNP at 48 hours from baseline	Percentage change in NT-proBNP at 48 from baseline
To compare the effect of a natriuresis guided diuretic strategy versus standard of care on percentage change in NT-proBNP at 72 hours from baseline	Percentage change in NT-proBNP at 72 from baseline

5.1.4 Safety endpoints

Safety endpoints include:

- SAE’s
- Renal Safety Events
  - Doubling of serum creatinine from baseline to 24 hours
  - Doubling of serum creatinine from baseline to 48 hours
- Pre-specified adverse events:
  - Worsening heart failure during hospitalization, defined as
    - Addition or starting of inotropes or vasopressors
    - Mechanical ventilation
    - Palliative care due to progressive heart failure
    - Any intervention/treatment for heart failure that leads to prolonged hospitalization
  - True worsening renal function, defined as
    - Doubling of creatinine from baseline to 48 or 72 hours without evidence of decongestion, or urine production < 10 cc / hour despite adequate dosing of loop diuretics

**Safety Endpoints:**	**Outcome Measure:**
To evaluate the safety and tolerability of a natriuresis guided diuretic strategy versus standard of care	1) Serious Averse Events (SAEs)<br>2) Renal Safety Endpoint:<br>a. Doubling of serum creatinine from baseline to 24 or 48 hours<br>3) Pre-specified adverse events:<br>a. Worsening heart failure<br>b. True worsening renal function

5.1.5 Exploratory Endpoints

- Total 72 hour natriuresis (from 0-72 hours)
- Total 72 hour diuresis (from 0-72 hours)
- Net negative fluid balance at 6, 12, 24, 48 and 72 hours
- Incidence of Hypo/hyperkalemia in the first 72 hours.

Exploratory Endpoints:	Outcome Measure:
To compare the effect of a natriuresis guided diuretic strategy versus standard of care on total natriuresis after 72 hours	Total natriuresis (mmol) from randomization to 72 hours after randomization
To compare the effect of a natriuresis guided diuretic strategy versus standard of care on total diuresis after 72 hours	Total diuresis (mL) from randomization to 72 hours after randomization
To compare the effect of a natriuresis guided diuretic strategy versus standard of care on net negative fluid balance at 6, 12, 24, 48 and 72 hours	Fluid balance (Fluid Intake minus Diuresis) for each time interval after randomization
To compare the effect of a natriuresis guided diuretic strategy versus standard of care on change in body weight at 24, 48 and 72 hours	Weight at baseline, 24, 48 and 72 hours
To compare the effect of a natriuresis guided diuretic strategy versus standard of care on diuretic response at 24, 48 and 72 hours	Weight change per 1 mg Bumetanide (40 mg furosemide) at baseline, 24, 48 and 72 hours
To compare the effect of a natriuresis guided diuretic strategy versus standard of care on change in NTproBNP	Percentage change in baseline NTproBNP at 24, 48 and 72 hours after randomization
To compare the effect of a natriuresis guided diuretic strategy versus standard of care on changes in serum potassium levels and incidence of hypokalemia (< 3.5 mmol/L) or hyperkalemia (> 5.5 mmol/L)	Change in serum potassium levels up to 72 hours and the incidence of hypo- and hyperkalemia

5.2 Definitions

5.2.1 End of study date

The study will be finished when the last follow-up visit of the last enrolled patient (last patient, last visit) is completed. This will be 180±10 days after inclusion of the last patient.

5.2.2 Withdrawal of consent

Withdrawal of consent means withdrawal from the study and should only occur if the patient does not agree to any further assessment at all. No data after date of withdrawal of consent should be collected. Data collected on or prior to date of withdrawal of consent will be included in the analyses.

5.2.3 Cross-over from treatment allocation group

Cross-over from treatment allocation group does not mean exclusion from the study or withdrawal of consent. Optimally, in these patients the follow-up visit should be completed per protocol. Data from these patients will be included in the intention to treat analyses according to their allocated treatment group. Events are included irrespective of the timing of the event in relation to the moment of cross-over.

5.2.4 Vital status

Known vital status at the end of the study is defined known whether the patient is dead or alive at the follow-up visit.

5.2.5 Lost to follow-up

The term lost to follow-up will be limited to patients with unknown vital status at the end of the study as defined in section 2.2.4.

5.3 Study design

This is a pragmatic, randomized, controlled, open label study in patients presenting with acute heart failure requiring intravenous loop diuretics. In total 310 patients will be randomized at the start of intravenous treatment (inhospital) to standard of care of natriuresis guided treatment. In the natriuresis guided treatment arm, decongestive treatment will be adjusted based on the spot urine sodium value assessed at set time points. Patients will be followed for the duration of the hospitalization. Adverse clinical events, including all-cause mortality and heart failure rehospitalization will be assessed by a telephone call after 6 months.

5.3.1 Randomization

Subjects will be randomized 1:1 to natriuresis guided therapy or standard of care. Randomization will be executed using an internal random number generator in the Electronic Health Record (EHR, EPIC, Verona, WI, USA). Each patient will be randomly assigned to either treatment arm when a patient's chart is opened by an eligible physician. This allocation is maintained as fixed variable in the EHR, ensuring randomization is only carried out onceWhen treatment with intravenous loop diuretic therapy is started, based on this randomization number, the Epic electronic health record automatically chooses the correct treatment orders (Orderset) and diagnostic tests based on the randomization arm.

5.3.2 Blinding

The study has an open label design, which means both patient and physician will know which randomized treatment the patient has been allocated to. However, to prevent contamination and cross-over between treatment arms, physicians will be blinded entirely to all urinary

sodium measurements (timed collections as well as spot urinary sodium) in the standard of care arm. These measurements will however be carried out at the local laboratory (which ensures the same measurement of our primary endpoint of 24 hour natriuresis), but results will be withheld at the laboratory department and only after the 6 month follow up period has passed will be send to the EHR. Additional alerts are in place to ensure that physicians are instructed not to order (unblinded) urinary sodium assessment in the standard of care arm, unless there is an important medical reason.

5.3.3 Number of patients

Based on a previous observational study in acute heart failure patients, the mean 24 hours sodium excretion was 398±246 mmol. In this population, not 2 hours, but 6 hours measurements were available, and in these patients, 36% of patients had an insufficient response, defined as urinary sodium <90 mmol or urine output <900 mL at 6 hours. Assuming a 40% improvement in these 36% of patients, and a conservative 15% improvement in the remaining patients in their 24-hours urinary sodium excretion because of closer monitoring compared with standard of care, this will assume an overall 24% improvement in 24-hours urinary sodium excretion (0.36*1.40 + 0.64*1.15). Therefore, to obtain a power of at least 80% at a two-sided significance level of 0.025 (Bonferroni correction for a dual-primary endpoint), we calculated a sample size of 125 patients in each group would be sufficient for the primary endpoint of 24-hours natriuresis. To prevent being underpowered due to drop-out or missing data, which is expected to be higher than average in this pragmatic study design and given the delicate nature of urinary collections, we will increase enrolment by 10% therefore requiring 140 patients per group, and enrolling a total of 280 patients.
Based on this sample size, we will have 81% power with a two-sided significance level of 0.025 to detect a hazard ratio of 0.49 for the other dual-primary endpoint of all-cause mortality and heart failure rehopitalization at 6 months (a reduction in events from 38% to 21%). However also accounting for 10% missing follow-up data, we will increase the total number of patients to 310 (155 patients per group). After closing of the database, a CONSORT diagram will be produced for transparent status of the subject reporting.

6 Analyses sets

There will be two treatment regimens in this trial, namely natriuresis guided or standard of care. The efficacy analyses will follow the intention to treat (ITT) principle in assigning patients to treatment groups, i.e. patients will be analyzed as randomized. Safety analyses will also assign patients to the treatment group as randomized.

- <u>Full analysis set</u>: all patients who have been randomized will be included in the full analysis set. This is the primary analysis set for the primary and secondary outcome variables. Following the intention-to-treat principle, patients are analyzed according to the treatment group they have been assigned to at randomization.
- <u>Per protocol set</u>: this is a subset of the full analysis set which consists of all randomized patients in the full analysis set that remained in their allocated treatment group for at least the first 24-hours and had no major protocol deviations. Major protocol deviations are those affecting the primary endpoint analyses.
- <u>Safety analysis set</u>: all randomized patients who were at least two hours in their allocated treatment group. Patients will be analyzed according to treatment group received.

All protocol deviations will be evaluated before database lock to determine whether patients can be included in the above described analyses sets. The most important protocol deviations listed below will be summarized by randomized treatment group:

- Patients who were randomized but did not meet inclusion and exclusion criteria
- Patients who received treatment based on the other allocation group (i.e. crossed over)
- Patients who either received insufficient amount of diuretics (either dose or combination diuretic therapy) or too much diuretics (dose or combination diuretic therapy) according to protocol
- Accidental unblinding of urinary sodium values in standard of care arm

7 Endpoint variables

7.1 Dual-primary endpoint

The primary endpoint of the PUSH-AHF trial consists of two distinct dual-primary endpoints, namely (i) total 24-h natriuresis and (ii) the first occurrence of the combined endpoint of all-cause mortality or HF rehospitalization at 6 months. The second part of the endpoint (time from randomization to the first occurrence of HF rehospitalization) will be adjudicated by the endpoint adjudication committee (EAC). Patients who did not have an event will be censored at the date of follow-up visit. Heart failure rehospitalization is defined as a hospital admission with signs and symptoms of heart failure requiring treatment. Members of the EAC are provided a complete, blinded, endpoint package for each rehospitalization. The package is assessed by the EAC members. In case of discrepancy between the evaluation of EAC members regarding the same event, the case is further discussed with the aim of developing a consensus. If the committee is unable to reach a consensus, the endpoint will be established by the EAC chair.

7.2 Secondary endpoints

The secondary endpoints are included in a hierarchical testing sequence following the primary endpoint, and will be tested if one of the dual-primary endpoints is statistically significant (at p-value 0.025). For the secondary outcomes, a two-sided P-value of 0.05 will be used if both primary endpoints are met. If only one primary endpoint will be met, a two-sided p-value of 0.025 will be used. Length of index hospitalization is calculated as the date of discharge from the hospital minus the date of screening plus 1. Patients still in the hospital at day 60 will be censored at day 60. If patients die during the index hospitalization, the maximum length of stay (60 days +1) will be assigned.

8 Statistical analysis

8.1 Closed Testing Procedure

The statistical analysis will use a pre-specified closed testing procedure, including a pre-specified hierarchical ordering of primary and secondary analysis. For the primary analysis, the type I error will be controlled at the two-sided 0.025 level, please see for more information 8.2. For the secondary endpoint, the type I error will be controlled at the two-sided 0.05 level. Statistical significance will continue in pre-specified order until an endpoint is rejected at two-sided 0.05 level.

8.2 Analysis of the primary endpoint

The primary endpoint will be assessed in the intention to treat (ITT) population. The dual-primary endpoint will be total 24-hour natriuresis and first occurrence of all-cause mortality or heart failure rehospitalisation at 6 months. Following the Bonferroni correction used for the sample size calculation, the study will be deemed positive if one or both of the components of the dual-primary endpoint are positive (*P-value*<0.025).

24-hour natriuresis will be calculated and presented as mean +/- SD if normally distributed, or median (25th and 75th percentile) in the case of non-normal distribution. The between group difference will be tested using t-test if normally distributed, or Wilcoxon rank sum test if non-normally distributed. The effect of natriuresis guided treatment on long-term outcomes will be assessed using Cox regression (assuming proportional hazard assumption is met) for the between treatment difference. Kaplan-Meier estimates will be calculated and plotted.

For the presentation of baseline characteristics in both treatment arms, continuous variables will be presented as mean +/- SD, non-normally distributed variables as median (25th – 75th percentile), and categorical values as count (percentages).

8.3 Secondary endpoint(s)

Secondary outcomes will be tested in the hierarchial testing scheme in the prespecified order of the endpoints as mentioned in 5.1.2 and 5.1.3. If both primary outcomes are rejected at two-sided p-value 0.025, no P-values will be calculated for the secondary outcomes.
Changes in biomarkers, natriuresis at subsequent time points, and length of hospital stay will be assessed using t-test for normal distributed variables, and Wilcoxon rank sum test if non-normally distributed.

8.4 Subgroup Analysis

For explanatory purposes subgroup analysis for the primary endpoint will be conducted, to assess whether the treatment effect is modified by different baseline characteristics. The table below gives an overview of the subgroups that will be investigated and how they will be created:

Characteristic	Subgroup
Age (years)	≤ median, > median
Sex	Male, Female
Left ventricular ejection fraction (if known)	≤ 40%, > 40%
Baseline NT-proBNP (ng/L)	≤ median, > median
Baseline estimated Glomerular Filtration Rate	≤ median, > median
Cause of heart failure	Ischemic, non Ischemic
Outpatient dose of loop diuretic	≤ median, > median
Hyponatremia (mmol/L)	≤ 135, > 135
Hypokalemia (mmol/L)	≤ 3.5, > 3.5
Atrial Fibrillation	Yes, No
SGLT2 inhibitor use at baseline	Yes, No

Between group differences in the second part of the dual-primary endpoint will be tested with use of a Cox proportional hazards model with the inclusion of an interaction term.

8.5 Other study parameters

Adverse event rates, including doubling of serum creatinine at 24 or 48 hours, serious adverse events, as well as worsening heart failure will be analyzed using Fisher's exact tests, and presented as counts (percentages). To study differences in repeated assessments between treatment groups, such as natriuresis during hospitalization, repeated measures linear mixed effect modeling will be used.

8.6 Missing data

The proposed statistical approach assumed missing data mechanism to be missing at random. If the level of missing data for the primary outcome in the full analysis set exceed 10%, a sensitivity analysis to assess the robustness of the analysis of the primary outcome by means of multiple imputation technique will be performed for the primary endpoint.

8.7 Other sensitivity analysis

This trial has been initiated during the COVID-19 pandemic, therefore no sensitivity analysis for the effect of COVID on heart failure hospitalization was deemed necessary.

8.8 Interim analysis (if applicable)

Not applicable

8.9 Pre-specified sub-analyses

In addition to the primary, secondary and exploratory endpoints and analyses as indicated in this SAP we prespecify the following analyses to be conducted after the main study results have been published.

8.9.1 Clinical Outcome

In this analysis, focus will be on the effect of natriuresis guided therapy on hard clinical endpoints, during and after hospitalization. This includes (but not restricted to) all cause mortality, cardiovascular and non-cardiovascular mortality and (repeated) hospitalizations, including heart failure hospitalization.

8.9.2 Congestion Status

In this analysis, focus will be on signs and symptoms of congestion. Association between treatment allocation, changes in congestion status, including biomarkers and associated outcome will be assessed.

8.9.3 Renal Function

In this analysis, the association between randomized treatment allocation and (changes) in renal function will be evaluated, including relationship with other parameters such as outcome.

8.9.4 HF Phenotype (HFrEF, HFmrEF, HFpEF)

In this analysis, the association between randomized treatment allocation and outcomes will be evaluated in different phenotypes of HF and the interaction between phenotype and treatment effect will be studied.

8.9.5 Win Ratio

In this analysis, the effect of randomized treatment on a Win-Ratio will be evaluated. One of the proposed win-ratio's will be a composite hierarchical investigated endpoint, including all cause death, HF rehospitalization and 24h natriuresis.

8.9.6 Home Diuretic Use

In this analysis, considering the association between diuretic responsiveness and home diuretic use and dose, the association between randomized treatment allocation and home diuretic use with outcomes will be evaluated

8.9.7 Pharmacodynamic substudy

In a small subgroup of patients included in the main trial, sequential urinary (and serum) samples have been obtained (via additional informed consent). In this subgroup of patients, the short-term effect of intravenous diuretic bolus treatment on diuresis, natriuresis will be evaluated.

8.9.8 Renal Ultrasound substudy

In a subgroup of patients included in the main trial, sequential renal ultrasounds have been executed (via additional informed consent). The main focus of this substudy is to evaluate the effect of diuretic treatment and randomized treatment allocation on echographic markers of (renal) congestion

8.9.9 Combined Analysis with ESCALATE

In this pre-specified analysis, we aim to combine data from PUSH-AHF with data from ESCALATE (NCT04481919). ESCALATE is different randomized, blinded controlled trial where a diuretic strategy, based on natriuresis, is tested against standard of care. We aim to include patient level data to investigate the effect of natriuresis guided treatment on heart failure outcomes, including natriuresis, diuresis, congestion parameters and clinical outcome. We have been into contact with the principle investigators of ESCALATE, who agree on this pre-specified analysis of both trials.
