# Supplementary material for: Natriuresis-guided diuretic therapy in acute heart failure: a pragmatic randomized trial
Source: Nat Med. 2023 Aug 28;29(10):2625–32. doi: 10.1038/s41591-023-02532-z (PMC10579092; doi:10.1038/s41591-023-02532-z)
Supplement: Supplementary file 2 — Reporting Summary [file 41591_2023_2532_MOESM2_ESM.pdf]

nature portfolio

Corresponding author(s): J.M. ter Maaten

Last updated by author(s): Aug 1, 2023

Reporting Summary

Nature Portfolio wishes to improve the reproducibility of the work that we publish. This form provides structure for consistency and transparency in reporting. For further information on Nature Portfolio policies, see our Editorial Policies and the Editorial Policy Checklist.

Statistics

For all statistical analyses, confirm that the following items are present in the figure legend, table legend, main text, or Methods section.

n/a	Confirmed	
☐	☒	The exact sample size (*n*) for each experimental group/condition, given as a discrete number and unit of measurement
☐	☒	A statement on whether measurements were taken from distinct samples or whether the same sample was measured repeatedly
☐	☒	The statistical test(s) used AND whether they are one- or two-sided<br>*Only common tests should be described solely by name; describe more complex techniques in the Methods section.*
☐	☒	A description of all covariates tested
☐	☒	A description of any assumptions or corrections, such as tests of normality and adjustment for multiple comparisons
☐	☒	A full description of the statistical parameters including central tendency (e.g. means) or other basic estimates (e.g. regression coefficient) AND variation (e.g. standard deviation) or associated estimates of uncertainty (e.g. confidence intervals)
☐	☒	For null hypothesis testing, the test statistic (e.g. *F*, *t*, *r*) with confidence intervals, effect sizes, degrees of freedom and *P* value noted<br>*Give P values as exact values whenever suitable.*
☒	☐	For Bayesian analysis, information on the choice of priors and Markov chain Monte Carlo settings
☒	☐	For hierarchical and complex designs, identification of the appropriate level for tests and full reporting of outcomes
☒	☐	Estimates of effect sizes (e.g. Cohen's *d*, Pearson's *r*), indicating how they were calculated

Our web collection on statistics for biologists contains articles on many of the points above.

Software and code

Policy information about availability of computer code

Data collection	REDCap version 12.4.6
Data analysis	R version 4.0.2

For manuscripts utilizing custom algorithms or software that are central to the research but not yet described in published literature, software must be made available to editors and reviewers. We strongly encourage code deposition in a community repository (e.g. GitHub). See the Nature Portfolio guidelines for submitting code & software for further information.

Data

Policy information about availability of data

All manuscripts must include a data availability statement. This statement should provide the following information, where applicable:

- Accession codes, unique identifiers, or web links for publicly available datasets
- A description of any restrictions on data availability
- For clinical datasets or third party data, please ensure that the statement adheres to our policy

Anonymised participant data can be made available upon requests directed to the corresponding author. Proposals will be reviewed on the basis of scientific merit, ethical review, available resources and regulatory requirements. After approval of a proposal, anonymised data will be made available for reuse. A steering committee will have the right to review and comment on any draft manuscripts based on these data before publication.

Research involving human participants, their data, or biological material

Policy information about studies with human participants or human data. See also policy information about sex, gender (identity/presentation), and sexual orientation and race, ethnicity and racism.

Reporting on sex and gender	Sex was determined based on self reported data. We have included prespecified subgroup analysis based on sex.
Reporting on race, ethnicity, or other socially relevant groupings	Race was collected based on self reported data. This trial was executed as a single center study in the north of the Netherlands, where the majority of the population is White; this is reflected in the enrolled trial population (>90% White patients). We were therefore unable to perform subgroup analyses based on this race.
Population characteristics	Patients with acute heart failure requiring treatment of intraveous loop diuretics were included in the trial. 45% of the enrolled population was female. The median age was 74 years.
Recruitment	Patients were recruited at the emergency department. In all patients presenting to the emergency department requiring treatment with iv diuretics, the treating physician was prompted to enroll the patient in the PUSH-AHF trial. Patients couldn't be included if they were admitted to a non-cardiology ward as these were not trained for the 24-hour collections and trial protocol. As the recruitment and randomization was incorporated in the electronic health record, all cardiology patients requiring iv diuretics at the emergency department were screened. If the treating physician thought the patient to be ineligible or there were logistical issues, the decision could be made to exclude the patient. Overall, only 17% of all screened patients were not enrolled in the trial.
Ethics oversight	The ethics committee of the University Medical Center Groningen approved the trial protocol.

Note that full information on the approval of the study protocol must also be provided in the manuscript.

Field-specific reporting

Please select the one below that is the best fit for your research. If you are not sure, read the appropriate sections before making your selection.

☒ Life sciences ☐ Behavioural & social sciences ☐ Ecological, evolutionary & environmental sciences

For a reference copy of the document with all sections, see nature.com/documents/nr-reporting-summary-flat.pdf

Life sciences study design

All studies must disclose on these points even when the disclosure is negative.

Sample size	310 patients were enrolled based on the following sample size calculation: A statistical power of 80% on mean change in total 24 hour natriuresis at day 1 at a two-sided significance level of 0.025 (Bonferroni correction for the dual primary endpoint) was ensured if 125 patients were available for this primary endpoint. For the second part of the dual primary endpoint (the combined endpoint of all-cause mortality or HF rehospitalisation at 180-days), with 140 patients per group with available data, a statistical power of 81% again at a two-sided significance level of 0.025 was available to detect a hazard ratio (HR) of 0.49.
Data exclusions	Patients with missing data (considered at random) for the first part of the dual primary endpoint were excluded for these analysis.
Replication	We found that the results were consistent in the predefined per protocol analyses.
Randomization	Patients were randomized to natriuresis guided therapy and standard of care, aiming to randomly assign 50% of patients to each group.
Blinding	Physicians and investigators were not blinded to the treatment allocation. As by design the spot urinary sodium values were required to adjust therapy in the natriuresis guided group, the treating physicians could not be blinded to this. The natriuresis values in the standard of care group were however blinded.

Reporting for specific materials, systems and methods

We require information from authors about some types of materials, experimental systems and methods used in many studies. Here, indicate whether each material, system or method listed is relevant to your study. If you are not sure if a list item applies to your research, read the appropriate section before selecting a response.

Materials & experimental systems

n/a	Involved in the study
☒	☐ Antibodies
☒	☐ Eukaryotic cell lines
☒	☐ Palaeontology and archaeology
☒	☐ Animals and other organisms
☐	☒ Clinical data
☒	☐ Dual use research of concern
☒	☐ Plants

Methods

n/a	Involved in the study
☒	☐ ChIP-seq
☒	☐ Flow cytometry
☒	☐ MRI-based neuroimaging

Clinical data

Policy information about clinical studies

All manuscripts should comply with the ICMJE guidelines for publication of clinical research and a completed CONSORT checklist must be included with all submissions.

Clinical trial registration	NCT04606927
Study protocol	The study protocol has been included in the submission.
Data collection	Data collection was incorporated in the electronic health record and consequently entered into REDcap
Outcomes	This study had a dual primary endpoint of 24-hour natriuresis and 180-day all-cause mortality or heart failure rehospitalization. Heart failure rehospitalization was adjudicated by an endpoint committee blinded to the treatment allocation.
